# Application of stimuli-responsive nanomedicines for the treatment of ischemic stroke

**DOI:** 10.3389/fbioe.2023.1329959

**Published:** 2024-02-02

**Authors:** Yongyi Zhan, Yue Dai, Zhejing Ding, Mingtian Lu, Zehua He, Zhengwei Chen, Yongkang Liu, Zhongliang Li, Guangsen Cheng, Shaojun Peng, Yu Liu

**Affiliations:** ^1^ Zhuhai Interventional Medical Center, Cerebrovascular Diseases Department, Zhuhai Clinical Medical College of Jinan University (Zhuhai People’s Hospital), Zhuhai, China; ^2^ Zhuhai Institute of Translational Medicine, Zhuhai Clinical Medical College of Jinan University (Zhuhai People’s Hospital), Zhuhai, China

**Keywords:** ischemic stroke, stimuli-responsive, nanomedicine, micro-environment, drug delivery

## Abstract

Ischemic stroke (IS) refers to local brain tissue necrosis which is caused by impaired blood supply to the carotid artery or vertebrobasilar artery system. As the second leading cause of death in the world, IS has a high incidence and brings a heavy economic burden to all countries and regions because of its high disability rate. In order to effectively treat IS, a large number of drugs have been designed and developed. However, most drugs with good therapeutic effects confirmed in preclinical experiments have not been successfully applied to clinical treatment due to the low accumulation efficiency of drugs in IS areas after systematic administration. As an emerging strategy for the treatment of IS, stimuli-responsive nanomedicines have made great progress by precisely delivering drugs to the local site of IS. By response to the specific signals, stimuli-responsive nanomedicines change their particle size, shape, surface charge or structural integrity, which enables the enhanced drug delivery and controlled drug release within the IS tissue. This breakthrough approach not only enhances therapeutic efficiency but also mitigates the side effects commonly associated with thrombolytic and neuroprotective drugs. This review aims to comprehensively summarize the recent progress of stimuli-responsive nanomedicines for the treatment of IS. Furthermore, prospect is provided to look forward for the better development of this field.

## 1 Introduction

### 1.1 What is ischemic stroke?

Ischemic stroke (IS) refers to a medical emergency in which brain cells are damaged or killed due to reduced blood flow to the brain tissue. The reduced blood flow is caused by cerebral artery stenosis, a thrombus in the cerebral artery, or a detached thrombus from other parts of the body. The occurrence of IS involves several risk factors, including hypertension, diabetes, smoking, dyslipidemia, obesity, and hyperhomocystinemia, among others (Gu et al., 2019; Cao et al., 2021; Walter, 2022; Wang et al., 2022). At present, the Trial of Org 10172 in Acute Stroke Treatment (TOAST criteria) is extensively applied in clinical practice. It analyzes the etiology of patients with IS based on the TOAST criteria, which considers large-artery atherosclerosis (LAA), cardioembolism (CE), small-vessel disease, lacunar (Lac) stroke, cryptogenic (Cry) stroke, and other causes (Arsava et al., 2017; Zhang et al., 2019). Ischemic necrosis of brain tissue in different areas can lead to a variety of symptoms. Common clinical manifestations include sudden unilateral numbness or weakness, slurred speech, visual disturbances, and vertigo.

Statistically, the incidence of stroke has declined in developed countries. However, stroke still remains the second leading cause of death worldwide and the primary cause of acquired disability, imposing a significant economic burden on all countries (Herpich and Rincon, 2020). In recent years, the prevalence of stroke patients has significantly increased. IS accounts for approximately 70%–80% of all stroke cases. While the mortality rate of IS has generally stabilized due to advancements in medical care, it has become as a major cause of death and disability in China (GBD, 2019 Stroke Collaborators, 2019; Wu et al., 2019; Chao et al., 2021; Wang et al., 2022; Tu et al., 2023). Therefore, there is an desperate demand to exploit innovative approaches for treating IS to prolong patient survival and improve their quality of life.

### 1.2 Pathophysiology of ischemic stroke

In recent years, there has been increasing recognition of the “neurovascular unit (NVU)” concept in the discussion of stroke pathophysiology. The NVU is an integrated functional unit consisting of endothelial cells, perivascular neurons, and astrocytes surrounding blood vessels. It highlights their collective role in cerebral blood flow instead of acting independently as individual units (Tiedt et al., 2022). As a result, IS can lead to a complex pathophysiological response, resulting in neuronal damage. There is currently a consensus on the key mechanisms of ischemic injury. These mechanisms include excitotoxicity, mitochondrial dysfunction, oxidative stress, inflammatory response, disruption of the blood-brain barrier (BBB), and cellular death (George and Steinberg, 2015; Tuo et al., 2022b; Qin et al., 2022). The damage caused by these initial three mechanisms can initiate diverse cell signaling cascades, leading to either programmed or non-programmed cell death in brain cells. The aforementioned complex processes collectively contribute to altering the microenvironment of ischemic tissue. These alterations encompass excessive generation of reactive oxygen species (ROS), energy depletion, imbalances in acid-base levels, aggregation of immune cells, and enhanced production of coagulation factors.

ROS are chemically active oxygen-containing molecules. The primary oxidant forms in ischemic brain tissue are hydrogen peroxide (H_2_O_2_), superoxide anions (· $O_{2}^{-}$ ), and hydroxyl radicals (·OH). After the onset of IS, cerebral blood flow decreases, leading to a reduction in oxygen and glucose supply. Consequently, there is an excessive generation of ROS through various pathways. A major source of ROS in this process is nicotinamide adenine dinucleotide phosphate oxidase 2 (NOX2). Based on the experimental findings of Ye et al. (Yingze et al., 2022), there is a significant increase in the generation of NOX2 and ROS at 3 days post IS, which gradually decreases from 3 to 14 days. Additionally, the ROS resulting from mitochondrial dysfunction can contribute to reperfusion injury and cell death in ischemic conditions.

In hypoxic conditions, the inhibition of mitochondrial oxidative phosphorylation occurs. Cells respond by temporarily switching to anaerobic glycolysis to sustain adenosine triphosphate production. The imbalance between oxidative phosphorylation and anaerobic glycolysis in the ischemic penumbra leads to a decline in extracellular pH.

In ischemic micro-environments, the major contributors to BBB dysfunction are the production of matrix metalloproteinases (MMPs) and myeloperoxidase (MPO), along with cellular abnormalities within the NVU. These disruptions are marked by the degradation of junction proteins and an elevation in permeability (Spitzer et al., 2022). The disruption of BBB function results in the infiltration of prothrombin from the bloodstream into brain tissue. In a study conducted by Tuo et al., 2022a, proteomic analysis was performed on post-ischemic tissues which revealed that prothrombin (stroke/control ratio = 10.88) exhibited the highest upregulation among the proteins analyzed.

### 1.3 Current treatment

During the acute stage of IS, the primary treatment focuses on achieving vascular recanalization and minimizing brain cell damage. Vascular recanalization therapies include the administration of thrombolytic drugs and endovascular therapy (EVT). The main objective of these therapies is to restore blood flow in ischemic brain tissue. Timely and proactive clinical interventions play a crucial role in improving the prognosis of patients with IS. After a comprehensive evaluation of indications and contraindications, specific individuals may meet the criteria for intravenous thrombolysis and/or EVT. EVT is especially appropriate for patients with large vessel occlusion, as it has been associated with improved functional outcomes. However, it is essential to note that such interventions also have the risk of adverse events, including cerebral hemorrhage. Additionally, many medical institutions that initially admit stroke patients may not have advanced interventional treatment options (Berkhemer et al., 2015; Albers et al., 2018; Yang et al., 2020; Jovin et al., 2022; Yoshimura et al., 2022).

Common thrombolytic drugs include Alteplase (r-tPA), Tenecteplase, among others (Tsivgoulis et al., 2023; Yogendrakumar et al., 2023). r-tPA is currently the only thrombolytic drug approved by the Food and Drug Administration (FDA). Multiple large-scale trials have demonstrated that r-tPA can reduce the risk of long-term disability following IS. However, the utilization rate of intravenous thrombolysis is low, typically ranging from 3.2% to 5.2%, mainly due to strict limitations on the time window, indications, and contraindications. From 2019 to 2020, the overall rate of intravenous thrombolysis for acute IS in stroke center units in China was 5.64% (Ye et al., 2022). Additionally, r-tPA has limited targeting efficiency and reperfusion rates and is associated with a risk of cerebral hemorrhage of approximately 6% (Wang et al., 2022). Thrombolytic drugs can activate fibrinolysis in non-ischemic areas, leading to bleeding complications. As a result, achieving targeted and efficient drug delivery remains a significant challenge in thrombolytic therapy (Herpich and Rincon, 2020; Marko et al., 2022; Walter, 2022; Wang et al., 2022).

Neuroprotective drugs have been used in clinical practice to improve brain tissue tolerance to ischemia and minimize reperfusion injury (Fisher and Savitz, 2022; Parvez et al., 2022; Xiong et al., 2022). Numerous neuroprotective agents that target various mechanisms, particularly those that combat oxidative and nitrosative stress, have been discovered. Bisufenton and uric acid function as oxygen free radical scavengers, while Edaravone acts as an antioxidant. These compounds have shown considerable therapeutic potential in preclinical trials for IS. However, the lack of established strategies hampers the translation of promising neuroprotective agents from preclinical efficacy to effective stroke treatments (Chamorro et al., 2016). A multi-center randomized controlled trial demonstrated the efficacy of Nerinetide, a neuroprotective agent, in preclinical stroke models of ischemia-reperfusion. However, in clinical trials, Nerinetide did not significantly improve favorable clinical outcomes after endovascular embolectomy compared to the placebo group (Hill et al., 2020). Numerous neuroprotective agents have the potential to mitigate ischemia/reperfusion (I/R) injury. However, these drugs are frequently limited by their short half-lives, restricted BBB penetration, and ineffective targeting efficiency. Traditional antithrombotic and neuroprotective drug therapies are limited by safety concerns and inadequate targeting capabilities. Therefore, there is an urgent demand to address the clinical gaps in the treatment of IS.

### 1.4 Nanomedicine therapy

Nanomedicines represent a groundbreaking approach in the prevention, diagnosis, and treatment of various diseases. Due to their size-dependent characteristics and increased surface area, nanomedicines exhibit remarkable capabilities in the field of drug delivery. Nanomedicines accomplish targeted delivery to precise tissues or cells by fine-tuning their size, shape, and surface properties, which allows for the increased drug concentration at specific sites. Moreover, nanomedicines possess distinctive optical and magnetic properties, rendering them well-suited for diagnostic imaging and therapeutic interventions (Liu et al., 2022b; Parvez et al., 2022; Zhuang et al., 2022; Khizar et al., 2023).

Up to now, various nanomedicines have been applied in the treatment of IS, as shown in Table 1. According to Table 1, nanomedicines which are used for drug delivery to IS can be broadly classified into four categories: organic, inorganic, biomimetic, and composite nanomedicines. Organic nanomedicines, composed of organic molecules, offer various advantages including exceptional biocompatibility, high bioavailability, and easy fabrication. Furthermore, they usually do not generate toxic byproducts during degradation within the body. Inorganic nanomedicines exhibit greater stability, unique optical and magnetic properties, and a higher surface area to volume ratio compared to organic nanomedicines. However, their synthesis process typically requires sophisticated and precise conditions and methods. Biomimetic nanomedicines are designed and manufactured by imitating the structures, functions, and mechanisms found in biological systems. They can enhance drug stability, solubility, and absorption by modifying their physical and chemical characteristics, enabling targeted delivery and precise release of medications. Nevertheless, it is crucial to emphasize that safety remains a top priority when using biomimetic nanomedicines for drug delivery purposes. Composite nanomedicines can combine the advantages of organic materials and inorganic materials. A commonly encountered composite nanomedicine is the metal-organic frameworks (MOFs), comprising of a crystalline structure formed by metal ions and organic ligands. MOFs display a porous structure and a significant surface area-to-volume ratio, facilitating enhanced drug loading. Their porous structure and surface modifications offer a high degree of flexibility, enabling effective targeted drug delivery and controlled release (Alkaff et al., 2020; Zenych et al., 2020; Ma et al., 2021; Yu et al., 2022b; Lin et al., 2022; Cheng et al., 2023; Ruscu et al., 2023).

**TABLE 1:** Endogenous and exogenous stimuli-responsive system for drug delivery.

Response	Linker	Nanoparticles	Drug	Model	Year	Reference
ROS						
	Phenylboronic acid pinacol ester	Maleinimide-PEG-DSPE	NR2B9C	MCAO	2018	Lv et al. (Lv et al., 2018)
		C-PEG-LysB polymer	Rapamycin	tMCAO	2019	Lu et al. (Lu et al., 2019)
		β-cyclodextrin	Tempol	MCAO	2021	Yuan et al. (Yuan et al., 2021)
		2-diethylaminoethylen (DEAE)-dextran	18β-glycyrrhetinic acid	Photochemically induced cerebral infarction model	2022	Jin et al. (Jin et al., 2023)
		Cyclic oligosaccharide β-cyclodextrin	DL-3-n-butylphthalide	MCAO	2023	Yang et al. (Yang et al., 2023)
	Oxalate bond	HBA-OC-PEG_2000_	Rapamycin	MCAO	2021	Luo et al. (Luo et al., 2021)
		SAOR@Cur	Curcumin; Resveratrol; Angelica polysaccharide	tMCAO	2022	Su et al. (Su et al., 2022)
**pH**						
	Benzamide bond	Polyethylene glycol	uPA	pMCAO	2016	Cui et al. (Cui et al., 2016)
	Imide bond	Oxidized dextran	uPA	_	2018	Li et al. (Li et al., 2019)
		Methoxy poly (ethylene glycol)-block-poly (2-diisopropyl methacrylate)	Succinobucol	tMCAO	2021	He et al. (He et al., 2021)
	The protonation and charge among the polycations	Polyion complex	tPA	MCAO	2019	Mei et al. (Mei et al., 2019)
	PDPA segment	mPEG-b- P (DPA-co-HEMA)-Ce6	Rapamycin	tMCAO	2021	Cheng et al. (Cheng et al., 2021)
	Coulombic force	Hydroxyethyl starch	Smoothened agonist	tMCAO	2021	Yang et al. (Yang et al., 2021)
**Enzyme**						
sPLA_2_	sn-2 ester bond	PMP-inspired nanovesicle	Streptokinase	Carotid artery thrombosis model in mice	2017	Christa L. Pawlowski et al. (Pawlowski et al., 2017)
Thrombin	NH2onorleucineoTPRSFLoCo SH	Block copolymer	Glyburide	MCAO	2018	Guo et al. (Guo et al., 2018)
	Thrombin-cleavable peptide with a sequence of LTPRGWRLGGC	Acetal-modified dextran polymer	r-tPA; ZL006e	MCAO	2019	Xu et al. (Xu et al., 2019)
	Thrombin-responsive peptide (GGLVPRGFGG, pep)	MnO_2_ nanoplatform	uPA	FeCl_3_-induced carotid thrombosis model	2021	Zhang et al. (Zhang et al., 2021)
Matrix metalloproteinase	The MMP-cleavable peptides and MMP-inactive peptides	Polyelectrolyte complex NPs	Stromal derived factor-1α; Basic fibroblast growth factor	Photothrombotic ischemic stroke	2018	Jian et al. (Jian et al., 2018)
**Light**						
	Au	Janus polymeric motors	Heparin	_	2018	Shao et al. (Shao et al., 2018)
	DPPT-BTTPE	Photothermal-activatable liposome	tPA	Photothrombotic ischemia model	2021	Cai et al. (Cai et al., 2022)
	Thermosensitive phospholipid (DPPC)	Ultrasmall gold nanorods	uPA	_	2021	Ahmed Refaat et al. (Refaat et al., 2021)
	Platelet membrane vesicle (PM)	Melanin nanoparticle	tPA	Rat carotid artery thrombosis model	2021	Yu et al. (Yu et al., 2022c)
**Magnetic field**						
	γ-Fe_2_O_3_	γ-Fe_2_O_3_ magnetic nanoparticle	L-arginine	Mouse model of cerebral, cortical ischemic stroke	2020	Li et al. (Li et al., 2020)
	IONP	Magnetic nanovesicles using IONP-harbored MSC	_	tMCAO	2020	Han Young Kim et al. (Kim et al., 2020)
	γ-phase iron oxide nanoring	Ferrimagnetic vortex-domain iron oxide nanoring (FVIO)	MSC	MCAO	2022	Liu et al. (Liu et al., 2022a)
**Ultrasound**						
	Mechanical vibration of the gel shell	Hollow nanogels	uPA	MCAO	2017	Teng et al. (Teng et al., 2018)
	_	Sub-micrometric CaCO_3_ –templated polymer capsules	r-tPA	_	2019	Clara Correa-Paz et al. (Correa-Paz et al., 2019)
	Sonosensitizer protoporphyrin IX (PPIX)	Platelet hybrid microglia platform	Interleukin-4 (IL-4)	MCAO	2020	Li et al. (Li et al., 2021b)

*NPs, Nanoparticles; MCAO, Middle cerebral artery occlusion; tMCAO, Transient middle cerebral artery occlusion; pMCAO, Permanent middle cerebral artery occlusion; uPA, Urokinase-type plasminogen activator; t-PA, Tissue plasminogen activator; r-tPA/rtPA, Recombinant tissue plasminogen activator; sPLA2:Secreted phospholipase A2; PMP, Platelet microparticle; IONP, iron oxide nanoparticle; MSC, Mesenchymal stem cell.

**SCHEME 1:**
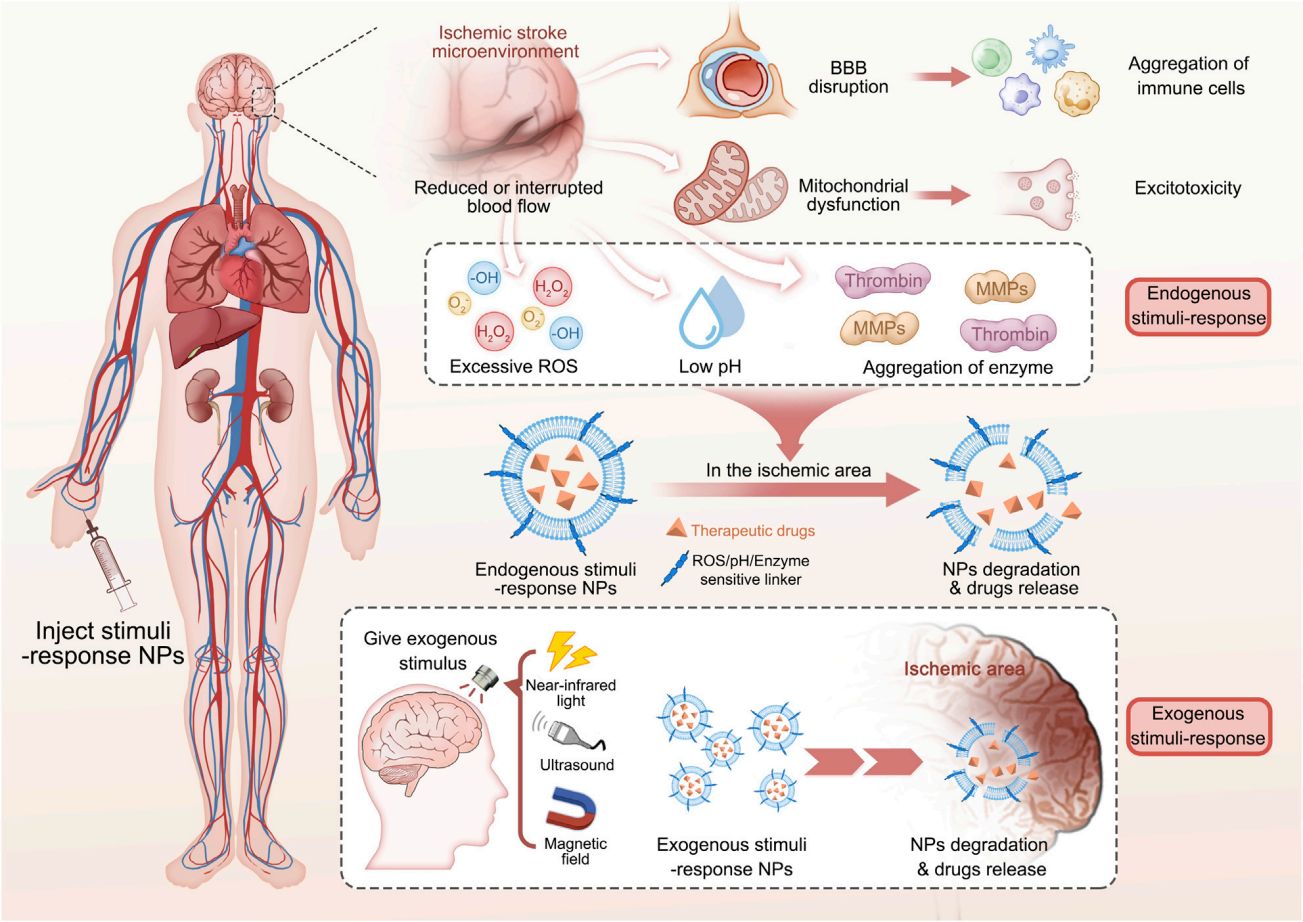
Endogenous and exogenous stimuli-responsive nanomedicines for the treatment of ischemic stroke.

In the treatment of IS, nanomedicines can be used to achieve controlled drug release, which is essential for maximizing therapeutic efficacy and ensuring *in vivo* safety. By carefully selecting appropriate nanomedicines and precisely manipulating their structures and properties, various methods can be utilized to achieve desired outcomes in drug release. In recent years, extensive research has shown that stimuli-responsive nanomedicines have the potential to greatly enhance therapeutic outcomes. These nanomedicines are designed to release payloads in response to either endogenous or exogenous stimuli. Endogenous stimuli-responsive nanomedicines achieve targeted drug release based on the pathophysiological conditions of ischemic brain tissue. These conditions include activated thrombin, excessive generation of ROS, and decreased pH levels in the ischemic tissues. By concentrating drug levels at the site of IS, this approach enhances treatment efficacy while minimizing adverse effects. Exogenous stimuli-responsive strategies use external stimuli such as magnetic fields, light, and ultrasound to precisely control the accumulation of nanomedicines in ischemic brain tissue. The targeted approach facilitates efficient drug delivery to the affected area, leading to controlled release, improved drug bioavailability, lower dosage and dosing frequency, effective therapeutic outcomes, and reduced drug-related side effects (Hassanpour et al., 2020; Li et al., 2021a). This review summarizes the recent research progress of nanomedicines in the field of IS therapy and analyzes their functional mechanisms (Scheme 1).

## 2 Endogenous stimuli-responsive nanomedicines

### 2.1 ROS stimuli-responsive nanomedicines

Specific pathophysiological changes occur in the IS microenvironment, and multiple mechanisms can induce ROS production or worsen oxidative stress injury. You et al., 2023 confirmed that the cellular ROS levels of experimental group increased rapidly to 1.8 times than that of the control group *in vitro* experiments using an oxygen-glucose deprivation model. Furthermore, an excessive generation of ROS following reperfusion therapy has the potential to induce reperfusion injury, thereby contributing to an unfavorable prognosis. Therefore, clearing ROS effectively and limiting oxidative stress are crucial in preventing extensive cytotoxicity caused by ischemia or reperfusion injury. Considering this pathophysiological mechanism, researchers have developed ROS-responsive nanomedicines consisting of ROS-responsive moiety, which can be broadly classified into four types: boric acid, carbonyl, sulfur or selenium, and proline oligomers (Saravanakumar et al., 2017; Liu et al., 2020; Zhao et al., 2020; Wang et al., 2021).

**FIGURE 1:**
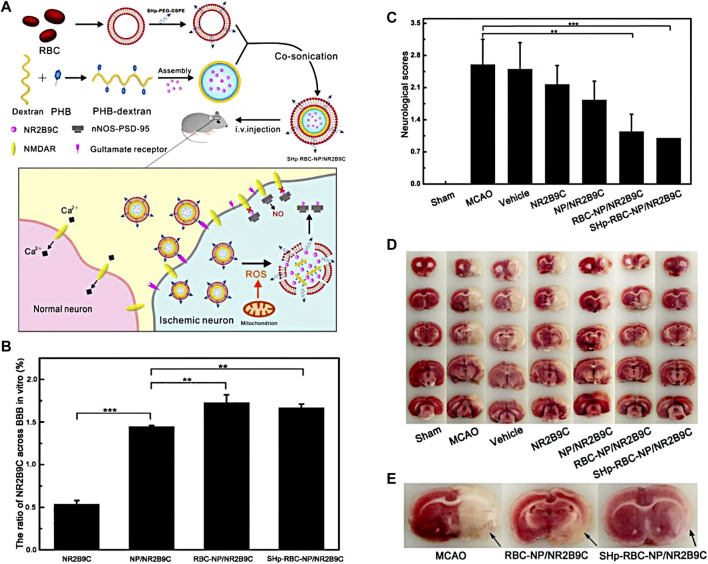
The fabrication of SHp-RBC-NP/NR2B9C and the results of animal experiments. **(A)** The fabrication of the nanomedicine. **(B)** The ratio of NR2B9C encapsulated in different samples transported to pass through BBB. **(C)** Neuroscores of rats after cerebral ischemia. **(D)** Representative slices of brain tissue from each experimental group. **(E)** Representative tissue slices demonstrating that the ROS-responsive nanomedicine greatly reduces the infarct volume. Reproduced with permission from ref (Lv et al., 2018). Copyright 2018, American Chemical Society.

Phenylboronic acid and its derivatives are primarily utilized as ROS-responsive groups for constructing targeted drug delivery systems in the treatment of IS. For instance, Lv et al., 2018 have developed a ROS-responsive nanomedicine containing with phenylboronic acid for IS treatment, as shown in Figure 1A. This nanomedicine utilizes the over-expressed ROS in the ischemic tissue as an intelligent “drug release switch,” which incorporates a ROS bio-reactive polymer named as glucan-conjugated phenylborate and utilizes borate-modified glucan polymer vesicles to load the neuroprotector NR2B9C. The surface of the vesicles is then encapsulated with red blood cell membranes (RBC) and modified with the stroke homing peptide (SHp). This nanomedicine could reduce the clearance by the reticuloendothelial system through the signaling molecule CD47 on the RBC, which extends its blood circulation and enables high-effective delivery to the ischemic injury area with the assistance of the SHp. Upon ROS stimulation, the phenylborate in the backbone of nanomedicines undergoes fast oxidation to form phenol and boric acid, resulting in the breakage of chemical bonds. As a result, the neuroprotector NR2B9C can be rapidly released into the IS tissue to facilitate neuronal repair (Figures 1B, C). *In vivo* experiments revealed that the group treated with the designed nanomedicine exhibited a significant decrease in cerebral infarction area and improved neurological deficits after ischemia-reperfusion (Figures 1D,E).

Enhanced mammalian target of rapamycin (mTOR) activity occurs during IS, and the signaling pathway contributes to ischemia-reperfusion injury. Inhibiting this pathway can exert a neuroprotective effect. Therefore, Lu et al., 2019 designed a polymeric micelle system called CPLB/RAPA which is utilized phenylborate as the ROS-responsive group. A fibrin-binding peptide called CREKA was conjugated to the micelle as a targeted group. Rapamycin (RAPA), an mTOR inhibitor, was loaded within the nanoparticles (NPs). In ischemic tissues, high-level of ROS facilitated the degradation of LysB, leading to the rapid release of rapamycin in local IS tissue. Therefore, CPLB/RAPA exhibits a direct neuroprotective effect by clearing ROS and the released rapamycin significantly inhibits the mTOR signaling pathway, which induces microglia polarization, protects the BBB, improves microvascular perfusion, and reduces brain tissue damage.

**FIGURE 2:**
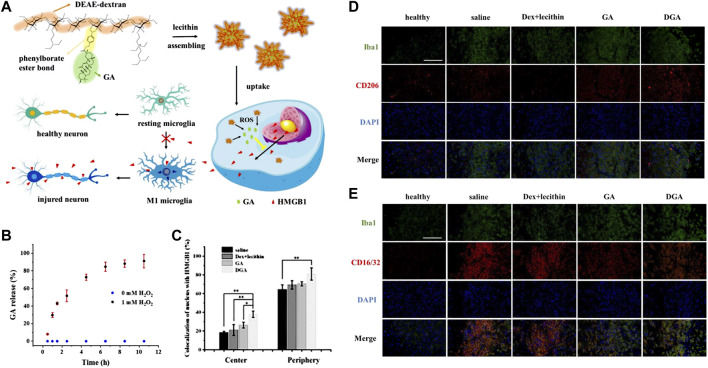
ROS-responsive DGA micelles for the treatment of IS. **(A)** The fabrication of DGA and its functional mechanisms. **(B)**The release behavior of GA in high-level of H_2_O_2_. **(C)** The quantitative nuclear localization percentages of HMGB1 measured in the central region of brain tissue injury. **(D)** Immunofluorescence staining on BV2 cells at the periphery of the injury site. CD206 was stained in red while Iba1 was stained in green. **(E)** Immunofluorescence staining was performed on BV2 cells at the periphery of the injury site. CD16/32 was stained in red. Reproduced with permission from ref (Jin et al., 2023).

In addition, Jin et al., 2023 recently fabricated ROS-responsive 18β-glycyrrhetinic acid (GA) conjugated diethylaminoethylen (DEAE)-dextran nanomedicines (DGA) by incorporating phenylborate into polymer backbones (Figure 2A). Phenylborate serves as the linkage between GA and glucan, which can be broken down by H_2_O_2_ in the oxidative stress environment of the IS. Consequently, GA can be released specifically in the focal area (Figure 2B). After stroke, high mobility group box 1 (HMGB1) exacerbates brain cell injury by participating in the neuroinflammatory cascade and promoting microglia polarization to the M1 phenotype. GA which is derived from glycyrrhizic acid significantly inhibits HMGB1 expression and phosphorylation (Figure 2C). Experiments have demonstrated that DGA effectively reduces brain tissue damage and provides protection to the ischemic penumbra (Figures 2D,E).

In a recent study conducted by Luo et al., 2021, a ROS-responsive amphiphilic copolymer named HBA-OC-PEG2000 (HOP) was developed, which was synthesized by chemically polymerizing hydroxybenzaldehyde (HBA) with oxalyl chloride (OC) and polyethylene glycol 2000 (PEG 2000). Rapa, as an effective drug for I/R injury, was loaded into HOP and subsequently encapsulated with a biomimetic cell membrane. This process led to the formation of RAPA@BMHOP, which is a promising approach. In the ischemic microenvironment, the OC in the NPs undergo rapid cleavage, leading to the fast release of RAPA. Owing to the anti-inflammatory, antioxidant, and neuroprotective effects, the accumulation of RAPA in IS tissue contributes to the relieving of the damage caused by ischemia and enhanced functional recovery following stroke. Additionally, Su et al., 2022 developed a type of nanomedicine named as MSAOR@Cur, which employed carbonyl groups as the ROS-responsive group and encapsulated with macrophage membrane to enhance the delivery efficiency. Notably, MSAOR@Cur exhibited a remarkable enhancement in post-stroke neurological function scores, thus offering a novel strategy for the neuroprotective treatment of stroke. You et al., 2023 developed ROS-responsive micelles loaded with luteolin and conducted *in vivo* experiments using the MCAO rat model. Under the high-level of ROS generated in the ischemic microenvironment, the thioketal bond of the polymer is cleaved, leading to the disintegration of the micelles. The release of luteolin from the micelles provides neuroprotective effects, leading to the superior treatment of IS.

In general, there is now much successful research for designing and synthesizing nanomedicines that can respond to high-level of ROS in the ischemic environment. The application of ROS-responsive nanomedicines for targeted delivery and controlled release of therapeutic drugs is a promising research field in the treatment of IS. However, large-scale clinical trials are still needed to further confirm their safety and efficacy. The results of these trials will determine whether ROS-responsive nanomedicines can become a clinically applicable treatment option. Therefore, continuous investment and effort are required in the research and development of this field to ultimately provide a safe and effective treatment for IS.

### 2.2 pH stimuli-responsive nanomedicines

After IS, the brain tissue experiences metabolic acidosis due to anaerobic glycolysis, ion imbalances and other factors. In a study conducted by Ilya V Kelmanson et al., 2021, it was found that the pH value in the core area dropped by approximately 0.5 units after the occurrence of an IS, ranging from 7.25 to 6.7. Furthermore, this decrease in pH level persisted for an extended period of time. The decreased pH level can activate cytokine receptors and inflammatory pathways, which further exacerbate brain tissue damage (Zhang et al., 2017). Recent studies suggest that pH can serve as a metabolic marker for distinguishing the ischemic core and penumbra following an IS. Nanotechnology offers promising prospects for utilizing pH-responsive NPs in delivering drugs to ischemic brain tissue (Harston et al., 2015; Leigh et al., 2018; Cheung et al., 2021). pH-responsive properties are typically attained through the utilization of chemical bonds that are stable at physiological pH value while susceptible to breakage at low pH values.

**FIGURE 3:**
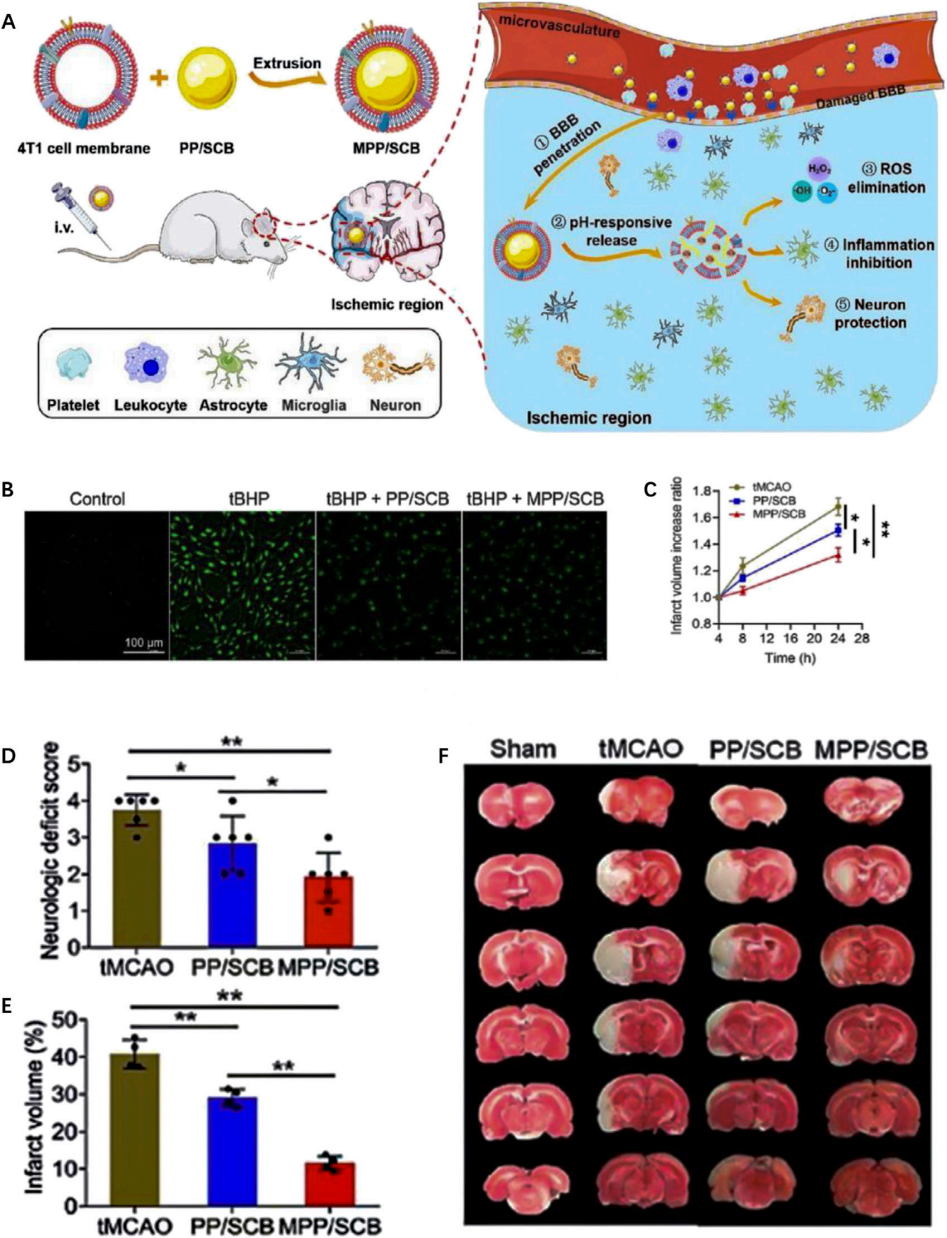
The preparation and therapeutic effect of pH-responsive MPP/SCB nanomedicine. **(A)** Scheme of the preparation and functional mechanism of MPP/SCB nanomedicine. **(B)** ROS-eliminating effects of each sample revealed by fluorescent image. **(C)** The increased ratio of brain infarct volume of tMCAO rats treated with different samples. **(D)** Neurological deficit scores of the tMCAO rats after different treatments. **(E)** Typical TTC staining images of the brain slices in sham-operated rats and tMCAO rats after different treatments. **(F)** Quantitative results of cerebral infarct volume after the treatments by different samples. Reproduced with permission from ref (He et al., 2021).

By utilizing benzamide bonds as pH-responsive sites, Cui *et al.* (Cui et al., 2016) developed a type of pH-responsive nanomedicine which is composed of polyethylene glycol and urokinase (PEG-UKs). It was observed that urokinase is released from PEG-UKs in acidic ischemic tissue due to the breakage of benzamide bonds in IS local environment. It is important to note that their findings do not assess the long-term efficacy of drug administration beyond the time window studied. However, they suggest that PEG-UKs have the potential to alleviate ischemic injury, although the precise underlying mechanism requires further elucidation. In a another study, Li et al., 2019 developed an RGD peptide-modified uPA-Oxd conjugate using imide bonds as the pH-responsive groups. In the acidic environment of ischemic brain tissue, the hydrolysis of imide bonds facilitates the fast release of uPA from the nanomedicine, thereby improving efficacy in thrombolytic treatment while reducing the risk of bleeding. In a related study conducted by He et al., 2021, pH-sensitive imines are incorporated into succinylbutylbutylene (SCB) glycol polymer and is camouflaged with the 4T1 cell membrane, resulting in the formation of a biomimetic nanomedicine (MPP/SCB) (Figure 3A). 4T1 cell membrane on the surface of MPP/SCB effectively facilitates the nanomedicine to across the BBB. ROS could be eliminated by the nanomedicine as shown in fluorescence imaging (Figure 3B). Of note, imine bonds are cracked in the acidic environment of IS tissue, leading to the rapid release of SCB. *In vivo* experiments demonstrated that MPP/SCB exhibits potent antioxidant and anti-inflammatory properties, which significantly optimizes the therapeutic outcomes of IS (Figures 3C–F).

In addition to the pH-triggered bond breakage, protonation behavior was also employed to fabricate pH-responsive nanomedicines in IS treatment. For instance, Cheng et al., 2021 developed pH-sensitive amphiphilic block copolymers with PDPA fragments. In the IS acidic environment, the hydrophobic PDPA fragments undergo a transition to hydrophilicity, leading to the expansion of the nanomedicines and subsequent rapid release of the loaded drugs. Furthermore, Yang et al., 2021 have developed pH-responsive dual-targeted NPs conjugated with an integrin ligand. Subsequently, they combined the negatively charged nanocarrier with a positively charged smoothened agonist (SAG) via pH-dependent electrostatic adsorption. *In vitro* experiments demonstrated that the release rate of SAG from NPs increases with decreasing pH, which can be attributed to the reduction in negative charge within the acidic environment of ischemic brain tissue. In a particular study by Zhang et al., 2022b, betulinic acid (BA) was selected as an antioxidant for promoting neuroprotection following a stroke. To ensure swift drug release under acidic conditions, they modified the carboxyl group at the edge of the BA ring to an amino terminal group through chemical conversion, resulting in the formation of betulamin (BAM). The results suggest that the acid responsiveness of the BAM is facilitated by the amino protonation process, which disrupts the nanoparticle structure, leading to the rapid drug release.

### 2.3 Enzyme-responsive nanomedicines

Following the occurrence of IS, various enzymes play a role in the pathophysiological processes, including thrombin and MMPs. Tuo et al., 2022a utilized lipid metabolomics, proteomics, and immunohistochemistry techniques to investigate this phenomenon. Through their research, they identified thrombin and its downstream product ACSL4, which is involved in arachidonic acid metabolism, as key proteins in the ferroptosis-induced cell death pathway during I/R. Moreover, the researchers analyzed serum samples collected from IS patients and compared them to a healthy control group. Surprisingly, proteomic analysis revealed no significant increase in thrombin levels within the serum of IS patients. This suggests that the observed substantial increase in thrombin within ischemic brain tissue is primarily produced in the brain itself rather than originating from the serum.

**FIGURE 4:**
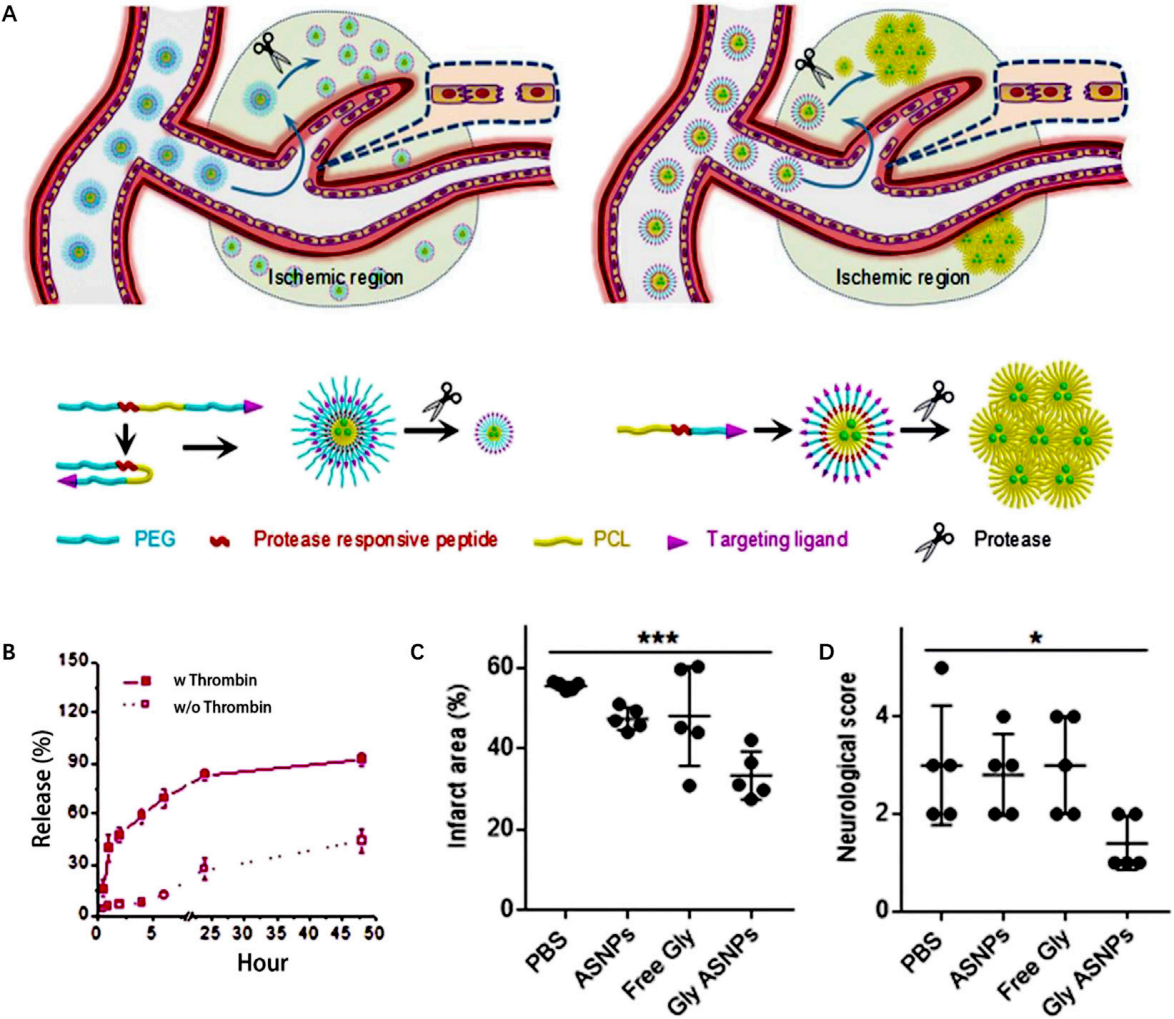
Enzyme-responsive polymeric micelles for enhanced anti-IS therapy. **(A)** The fabrication and functional mechanism of enzyme-responsive nanomedicines for anti-IS therapy. **(B)** Drug release profile with and without thrombin (100 nm). **(C)** The infarct area of MCAO mice after different treatment on postoperative day 3. **(D)** The neurological functional scores of MCAO mice after different treatment on postoperative day 3. Reproduced with permission from ref (Guo et al., 2018).

**FIGURE 5:**
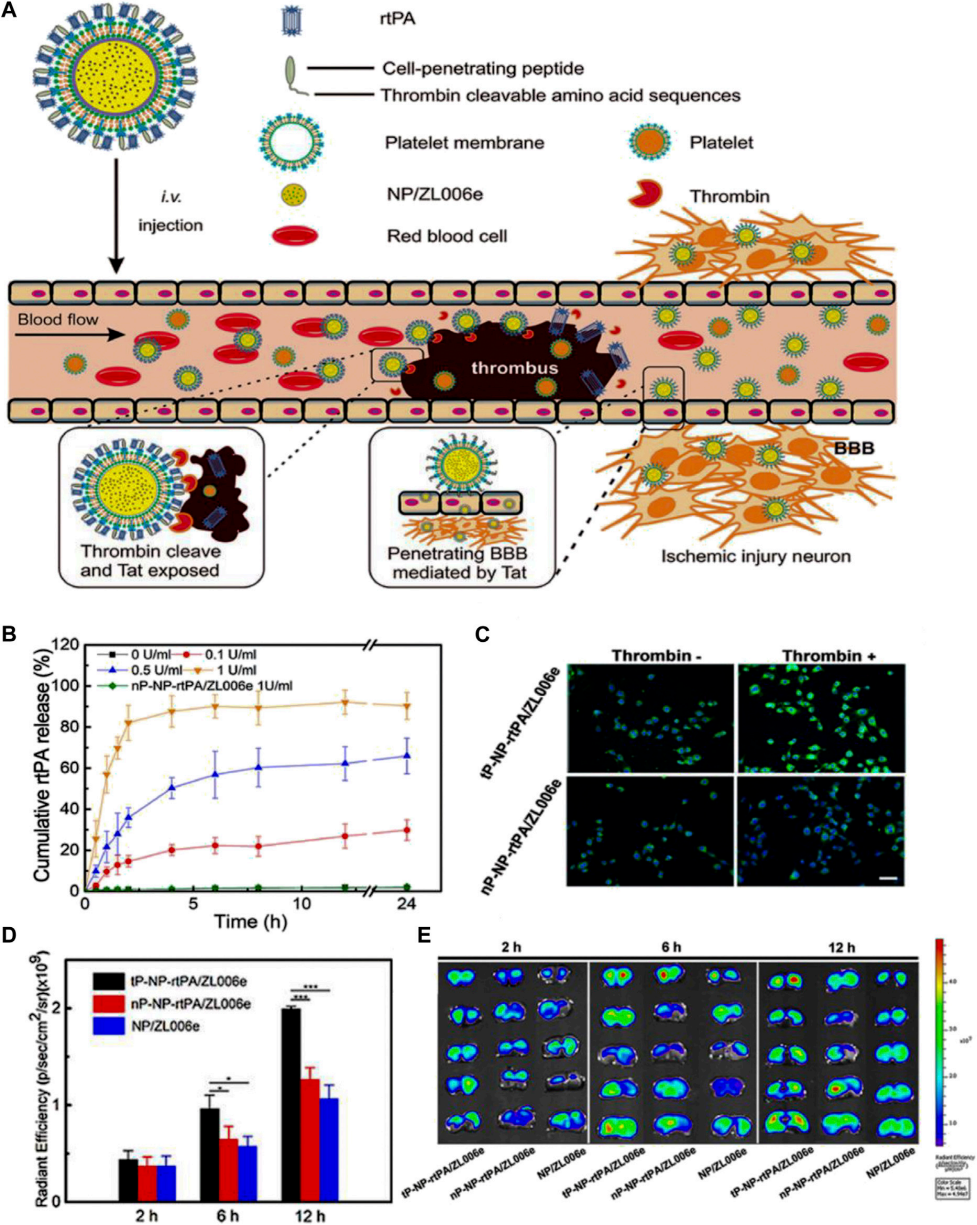
Enzyme-responsive biomimic nanomedicine for enhanced anti-IS therapy. **(A)** The fabrication and functional mechanism of enzyme-responsive biomimic nanomedicine for anti-IS therapy. **(B)** The release profile of rtPA from the nanomedicine in different level of thrombin. **(C)** The uptake behavior of the nanomedicine by BCEC cells with or without thrombin. **(D)** Fluorescence quantification of brain sections of different groups after systematic injection. **(E)** Fluorescence microscopy images of brain sections after systematic injection. Reproduced with permission from ref (Xu et al., 2019).

Christa L. Pawlowski et al., 2017 develop platelet-inspired NPs (PMINs) which are designed to release thrombolytic agents in response to the clot-relevant enzyme phospholipase-A2 (sPLA2). sPLA2 is produced by activating platelets and inflammatory cells in ischemic tissue, which could cleave the sn-2 ester bonds in distearyl phosphatidyl choline (DSPC). The cleavage destabilizes the lipid bilayer of the vesicle, resulting in the release of the encapsulated streptokinase (SK) contained within PMINs. *In vitro* experiments demonstrated the significant membrane degradation effect of sPLA2 on PMINs. The percentage of SK released by PMINs exposed to sPLA2 within the first 2 h was approximately four times higher than that of unexposed enzymes. Furthermore, *in vivo* experiments demonstrated that SK-loaded PMINs could effectively achieve thrombolysis while reducing off-target side effects on systemic hemostasis. Guo et al., 2018 developed a ligand-conjugated polymeric nanomedicines for the enhanced treatment of IS (Figure 4A). The nanomedicines were fabricated with polyethylene (PEG), poly (ε-caprolactone) (PCL), enzyme-cleavable peptides and ligands AMD3100 (CXCR4 antagonists). The peptides in the micelles were cleaved by high-level of thrombin in the ischemic environment, leading to the disruption of the micelle structure and enhances drug release to brain tissue (Figure 4B). *In vivo* results demonstrated that enzyme-responsive micelles achieved effective anti-IS therapy by enhancing local drug concentrations in IS tissue (Figures 4C,D). Moreover (Xu et al., 2019), developed a thrombin-responsive platelet biomimetic nanomedicine (named as tP-NP-rtPA/ZL006e). This nanomedicine is composed of a thrombin-cleavable peptide which is conjugated to a core of dextran derivative polymer and is coated with platelet membranes (Figure 5A). In the presence of thrombin, glyburide is accelerated released owing to the breakage of peptide in the nanomedicine (Figure 5B). *In vitro* experiments shows that the nanomedicine demonstrates enhanced uptake by BCEC cells with thrombin (Figure 5C). *In vivo* experiments revealed that tP-NP-rtPA/ZL006e significantly improved the anti-ischemic stroke treatment effect in the model of MCAO rats (Figures 5D, E).

Except for thrombin, MMPs also represent crucial enzyme targets for IS drug delivery. For instance, Jian et al., 2018 developed a nanohybrid hydrogel using hyaluronic acid (HA) hydrogel matrices which are incorporated with stromal derived factor-1α (SDF-1α) and basic fibroblast growth factor (bFGF). The hydrogel enables controlled release of nerve growth factor in IS, due to the high-level of MMP in ischemic brain tissue, which effectively cleaves the HA. In addition, Zhang et al., 2021 made modifications to the surface of MnO_2_ NPs by incorporating fucoidan (Fuco), a naturally occurring water-soluble polysaccharide, which is conjugated with a thrombin-reactive peptide (GGLVPRGFGG, pep). Additionally, urokinase-type plasminogen activators (uPA) were used as the therapeutic drug to create an enzyme-responsive nanomedicine for anti-IS therapy. Both *in vitro* and *in vivo* experiments confirmed the efficacy of the fabricated nanomedicine in achieving precise thrombolysis and remodeling of the neuroinflammatory microenvironment associated with stroke. What’s more (Wang et al., 2023), developed a peptide-based template PNzyme/MnO_2_ nano enzyme which utilizes the T7 sequence (HAIYPRH) and stroke homing sequence (CLEVSRKNC) to facilitate crossing of the BBB and accumulation in ischemic tissue. Upon recognition and cleavage by thrombin, the nanomedicine releases a thrombolytic peptide, initiating the process of thrombolysis. Moreover (Yu et al., 2022a), utilized a polyphenolic complex called tannin (TA) to establish non-covalent interactions between uPA, thrombin-cleavable peptides, and mesoporous silica templates. This approach allowed for the construction of a thrombin responsive nanodrug delivery system (NDDS) known as MS@uPA/PEP/TA NPs. By employing TA as a connecting agent, the uPA and thrombin-cleavable peptides were efficiently linked to the mesoporous silica templates. This design enables the NDDS to respond to thrombin in a controlled manner. The resulting MS@uPA/PEP/TA NPs hold promise for precise drug delivery and targeted therapy in the context of stroke treatment (Wu et al., 2023). designed the MMP-responsive NDDS using the sequence -GPLGIAGQ-as a cleavable peptide. Mesoporous polydopamine NPs were used as carriers and conjugated with RAP-12, a brain-targeting peptide on the surface. It was found that the NDDS could successfully deliver the neuroprotective agent, 9-aminominocycline, into the ischemic brain region. Importantly, the NDDS achieved triggered drug release by responding to the excessive level of MMP-2, which leaded to the enhanced therapeutic outcome.

## 3 Exogenous stimuli-responsive nanomedicine

Exogenous nanomedicines usually rely on the unique properties of metal inorganic NPs, such as magnetic and optical features, to respond to externally applied stimuli, including near-infrared light (NIR), magnetic fields, ultrasound, and so on. External stimulus response has the advantages of spatiotemporal control, precision, and convenience. The exogenous stimuli could facilitate targeted drug delivery and controlled release in IS local tissue, thereby resulting in improved efficacy of anti-IS treatment (Rahoui et al., 2017; Zhao et al., 2019).

### 3.1 Light stimuli-responsive nanomedicine

Recently, photothermal effect has been found to exert excellent anti-thrombotic effect in anti-IS therapy. Upon NIR irradiation, heat is generated locally in IS tissue, which could effectively disrupt the non-covalent interaction of fibrin, leading to the dissolution of blood clots (Cook and Decuzzi, 2021). Numerous studies have suggested the therapeutic potential of photothermal induced thrombolysis in the treatment of thrombotic diseases. In addition to the thrombolytic properties, researchers have also proposed that NIR radiation may have a neuroprotective effect in animal models of ischemia (Gerace et al., 2021). Commonly utilized NPs in the field include a variety of metal inorganic nanomaterials like gold nanorods, as well as organic nanomaterials such as liposomes and micelles. In the realm of light triggered NDDS, several methods have been employed to control drug release. These include: 1) Inducing a temperature increase through light irradiation, which in turn triggers the cleavage of covalent bonds to facilitate drug release. 2) Exploiting light irradiation-induced structural changes in NPs.

**FIGURE 6:**
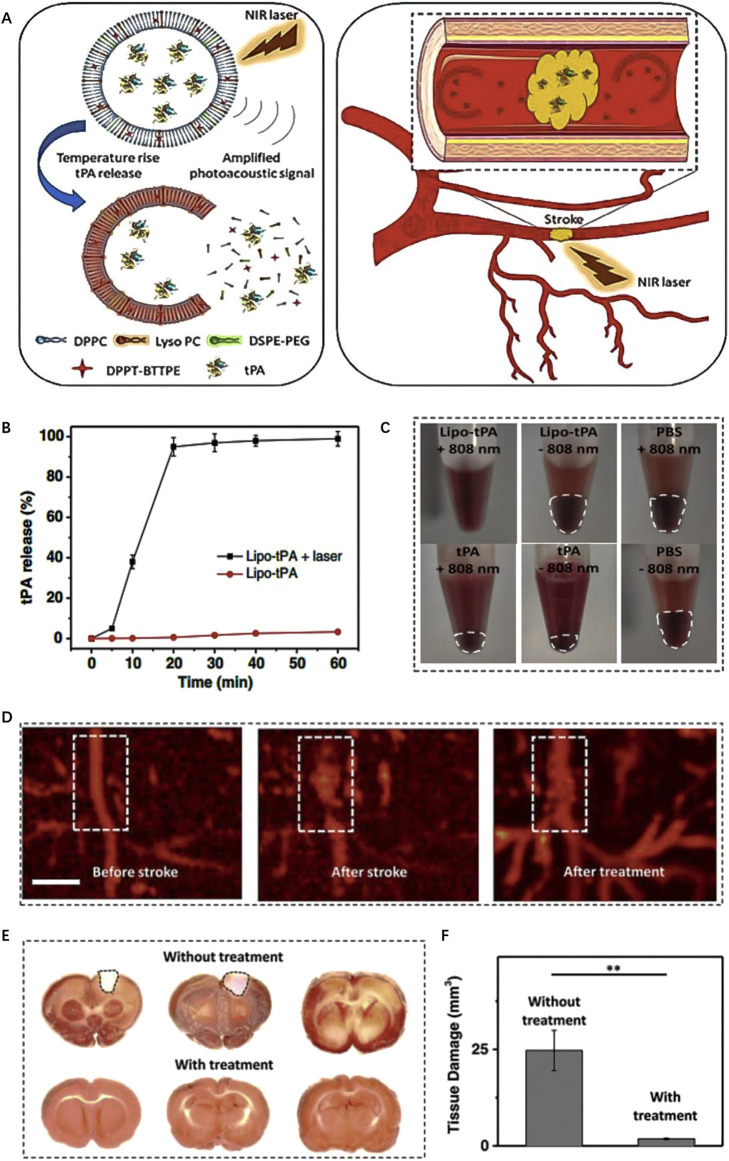
Enzyme-responsive biomimic nanomedicine for enhanced anti-IS therapy. **(A)** The fabrication and functional mechanism of enzyme-responsive biomimic nanomedicine for anti-IS therapy. **(B)** The release profile of rtPA from the nanomedicine in different level of thrombin. **(C)** The uptake behavior of the nanomedicine by BCEC cells with or without thrombin. **(D)** Fluorescence quantification of brain sections of different groups after systematic injection. **(E)** Fluorescence microscopy images of brain sections after systematic injection. Reproduced with permission from ref (Xu et al., 2019).

For instance, Shao et al., 2018 developed a type of light-responsive nanomotors, which harness the conversion of NIR light energy into mechanical kinetic energy, thereby generating autonomous motion. The researchers utilized chitosan (CHI) and heparin (Hep) to construct Janus capsules via layer-by-layer self-organization techniques. These capsules were then cloaked with RBC to enhance biocompatibility and partially coated with a layer of gold (Au) to confer NIR responsiveness. The asymmetrical Au coating of nanomotor leads to the generation of a temperature gradient when exposed to NIR laser irradiation. As a result, the nanomotor exhibit movement through an autophoretic effect, wherein the “on/off” switch is controlled by NIR stimulation. *In vitro* experiments demonstrate that nanomotor exhibits controllable motor performance under NIR irradiation and can effectively ablate thrombi through photothermal therapy. Besides, Cai et al., 2022 devised a photothermally activated liposome encapsulating tPA (Figure 6A). The liposome is composed with DPPT-BTTPE, which is an organic molecule with a propeller structure that exhibits proper absorption within the NIR range. The DPPT-BTTPE molecule serves dual roles: amplifying the photoacoustic signal and regulating the localized temperature rise that occurs due to the photothermal effect. This control ensures that the temperature remains within an optimal range, thus preventing tPA inactivation and potential tissue damage caused by excessive heating. Under NIR irradiation, the lipid bilayer of the liposome experiences heating and expansion, leading to the release of tPA (Figures 6B,C). Consequently, this system enables the controlled delivery of thrombolytic drugs specifically within the ischemic brain tissue, promoting effective thrombolysis, and minimizing the risk of thermal-related complications (Figures 6D–F).

Furthermore, Ahmed Refaat et al., 2021 developed a novel NIR-responsive liposomes consisting of gold nanorods (AuNRs), thermosensitive phospholipid (DPPC) and non-ionic surfactant (Brij58). When exposed to NIR light, the local temperature increases and the presence of Brij58 enables the disruption of the liposome membrane through the formation of stable nanopores, facilitating the efficient release of the encapsulated drug. In addition, Yu et al., 2022c devised a biomimetic nanovesicle termed tPA/MNP@PM (tMP), which is consisting of melanin NPs with high photothermal conversion efficiency, tPA and platelet membrane vesicles. Due to the thrombus-targeting and adhesive properties of platelet membrane, the nanovesicles exhibit effective localization at thrombus sites. Additionally, the NIR-mediated photothermal effect of tMP facilitates nanovesicle rupture, leading to precise release of tPA within the thrombus. Both *in vivo* and *in vitro* experiments have confirmed the therapeutic efficacy for IS. The study demonstrates the potential of biomimetic nanovesicles as a promising approach for targeted thrombus treatment, leveraging the benefits of thrombus specificity and controlled drug release under NIR-mediated photothermal effect.

### 3.2 Magnetic stimuli-responsive nanomedicine

**FIGURE 7:**
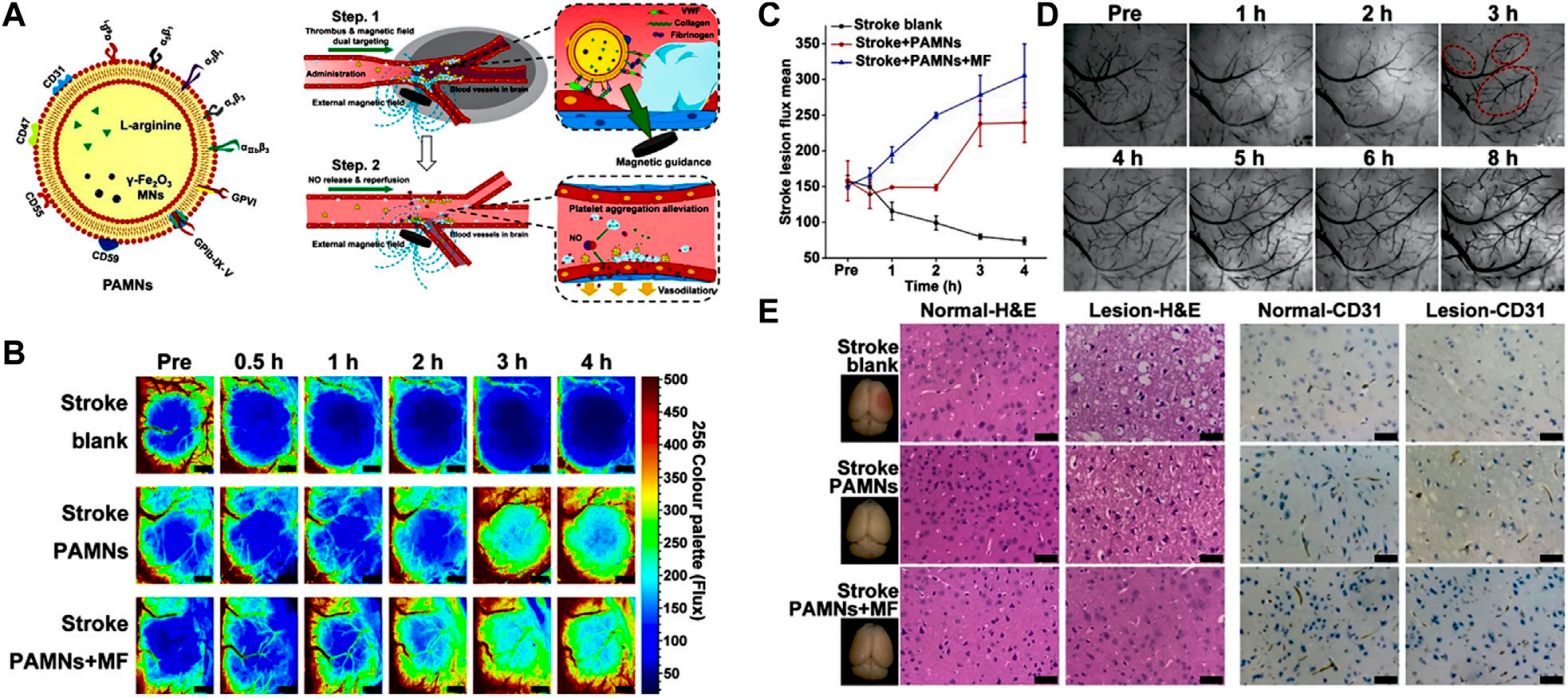
Magnetic stimuli-responsive platelet membrane biomimetic nanomedicine for anti-IS therapy. **(A)** The targeting mechanism of the designed nanomedicine *in vivo*. **(B)** The blood flow is observed by color-coded laser speckle images after different treatments. **(C)** Quantitative analysis of blood flow in ischemic lesions. **(D)** Bright field images of the stroke vascular vessel network. **(E)** H&E staining and CD31 staining for normal brain (left) tissue and ischemic lesion (right) tissue. Reproduced with permission from ref (Li et al., 2020).

Magnetic NPs offer significant potential as a platform for precise nanomedicine delivery, as they can be controlled and guided under external magnetic fields. To improve the stability and biocompatibility, magnetic NPs are usually coated with biocompatible polymers or encapsulated within lipid NPs. For instance, Hu et al., 2018 incorporated tPA into porous magnetic iron oxide microrods (tPA-MRs) for targeted thrombolytic therapy in IS. The magnetic properties of the NPs can be tailored based on the direction of the applied magnetic field. In a similar vein, Alba Grayston et al., 2022 functionalized poly (D-L-lactic acid-co-glycolic acid) (PLGA) with superparamagnetic iron oxide. The obtained NPs enabled magnetically targeted delivery of drugs to the IS local tissue. It is worth noting that Li et al., 2020 developed a type of platelet membrane biomimetic nanomedicine which is loaded with L-arginine and γ-Fe_2_O_3_ magnetic NPs (PAMNs) (Figure 7A). The magnetization of PAMNs was measured using a vibrating sample magnetometer (VSM), demonstrating excellent water solubility and superparamagnetic characteristics. The platelet membrane on PAMNs facilitated the effective across over BBB, due to its thrombus targeting ability. Under the stimulation by magnetic field, PAMNs could improve the blood flow effectively by the controlled release of L-arginine which generated NO in IS tissue to realize the significant vasodilatation (Figures 7B–D). *In vivo* experiments validated that magnetic stimuli-responsive platelet membrane nanomedicine could effectively relieve the stroke symptom, as shown in Figure 7E.

**FIGURE 8:**
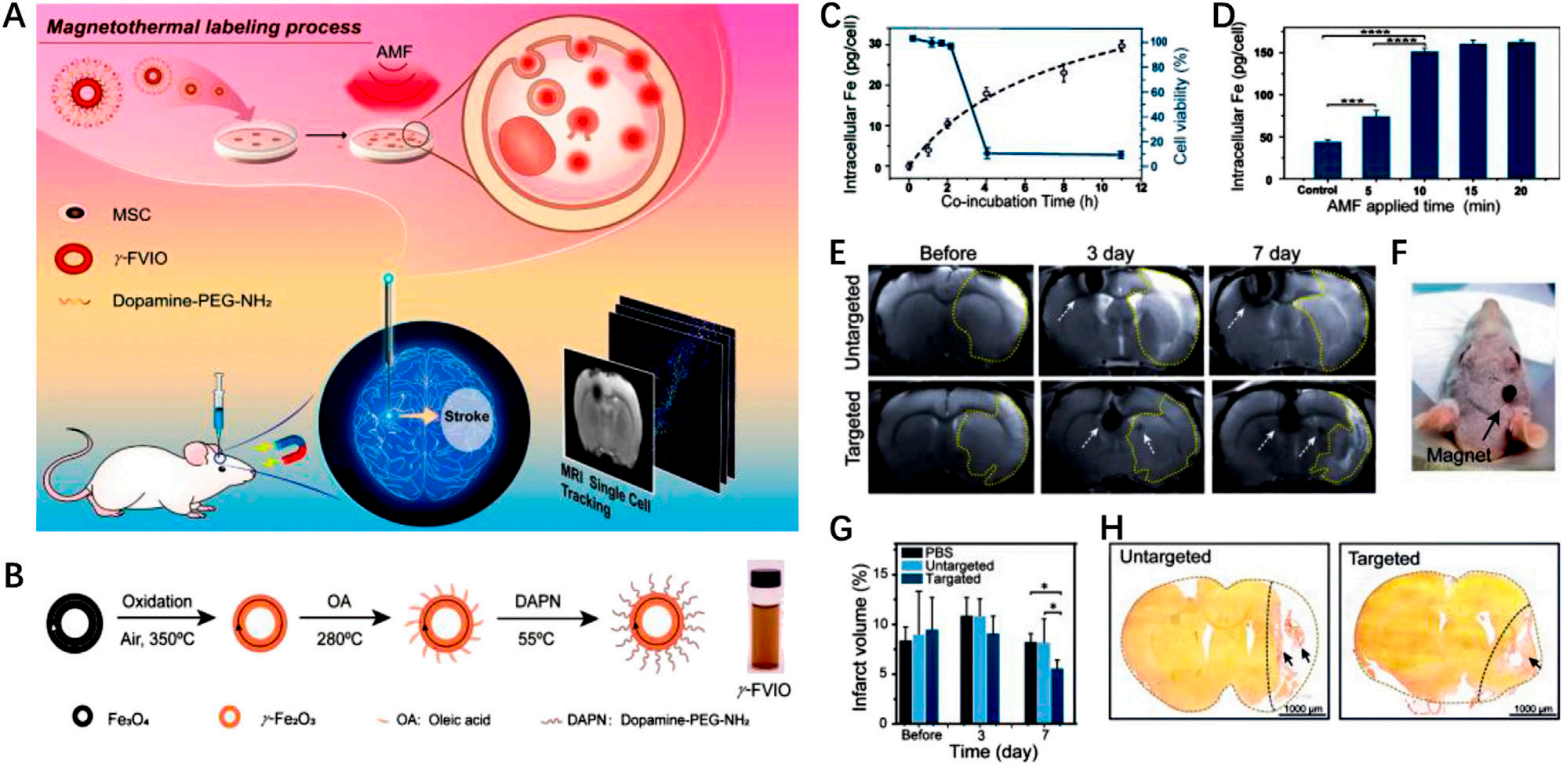
Magnetic stimuli-responsive mesenchymal stem cells-based nanomedicine for anti-IS therapy. **(A)** Scheme of the designed nanomedicine for MRI-guided imaging and therapy. **(B)** The fabrication process of γ-FVIO. **(C)** Intracellular uptake and cell viability of γ-FVIOs in different conditions. **(D)** Intracellular Fe content of MSCs at different time. **(E)** MRI imaging of cerebral infarct rats before and after treatment. **(F)** Photograph of magnetic targeting in rat brain. **(G)** The infarct volume of rats at different times. **(H)** The brain tissues rats with different treatments. Reproduced with permission from ref (Liu et al., 2022a).

Han Young Kim et al., 2020 developed magnetic nanovesicles (MNV) using iron oxide nanoparticle-harbored mesenchymal stem cells (MSC-IONP). The superparamagnetic nature of IONP allows them to be guided to ischemic areas in the brain under an applied magnetic field. The *in vitro* and *in vivo* experiments revealed that the utilization of a magnetic field resulted in a substantial increase in the accumulation of MNV in the cerebral ischemic tissue of a rat stroke model. The accumulation of MNV was observed to be approximately 5.1 times higher compared to the group that did not receive the magnetic field application. MNV treatment exerted its therapeutic effects through three pathways, including stimulating angiogenesis, preventing apoptosis, and promoting the differentiation of macrophages from an M1 to an M2 phenotype. In another study, Liu et al., 2022a utilized γ-Phase ferrimagnetic vortex-domain iron oxide nanorings (γ-FVIOs) for labeling mesenchymal stem cells (MSCs) for field-mediated targeted delivery (Figures 8A,B). Under magnetic field, the nanomedicine showed enhanced intracellular uptake by MSCs (Figures 8C,D). Furthermore, the superparamagnetic properties of γ-FVIOs enabled sensitive and sustained *in vivo* magnetic resonance imaging (MRI) tracking (Figures 8E,F). Both *in vitro* and *in vivo* experiments demonstrated the enhanced magnetic mobility of MSCs, enabling their rapid delivery to ischemic brain tissue for enhanced anti-IS therapy (Figures 8G,H). These studies highlight the potential of superparamagnetic NPs in facilitating targeted delivery of therapeutic agents to ischemic brain tissue, offering promising strategies for stroke treatment.

### 3.3 Ultrasound stimuli-responsive nanomedicine

Ultrasound possesses several advantages such as non-invasiveness, safety, ease of operation and excellent tissue penetration. Consequently, ultrasound-responsive nanomedicines have been widely used for targeted drug delivery. Ultrasound-responsive drug delivery primarily exploits the acoustic effects of ultrasound, which encompass mechanical and thermal effects. The mechanical effect involves the disruption of nanomedicine structures through acoustic radiation and cavitation effects to facilitate drug release. The thermal effect entails the conversion of ultrasound energy into heat, thereby raising the temperature within the target region, which causes structural changes in the nanomedicine to trigger drug release and increases vascular permeability to enhance drug accumulation (Zhao et al., 2013; Athanassiadis et al., 2022; Zhang et al., 2022a; Fan et al., 2022).

For example, Teng et al., 2018 synthesized uPA-loaded hollow nanogels (nUKs) comprised of glycol chitosan (GC) and benzaldehyde-capped polyethylene oxide. The experiments revealed that nUKs exhibited a responsive behavior to 2 MHz ultrasound. When exposed to ultrasonic energy, the cross-linked polymer matrix of nUKs underwent vibration, enabling the effective release of encapsulated uPA. To evaluate the therapeutic potential of nUKs in IS treatment, the researchers employed an IS rat mode, which demonstrated that nUKs, triggered by mechanical stress under ultrasound mediation, enhanced thrombolytic efficacy without increasing the risk of bleeding. Using the layer-by-layer method, Clara Correa-Paz et al., 2019 engineered nanocapsules (NCs) referred to as sub-micrometric CaCO_3_-templated polymer capsules with a diameter of approximately 600 nm, which were utilized to encapsulate r-tPA. *In vitro* experiments demonstrated that ultrasound-mediated delivery of r-tPA promotes thrombus decomposition. *In vivo* experiments revealed that a significant extension in the half-life of r-tPA when it is encapsulated within ultrasound-responsive NCs, suggesting enhanced thrombolytic efficacy under ultrasound stimulation. During an IS event, microglia usually exhibit two phenotypes known as M1 and M2. M1 microglia upregulate pro-inflammatory cytokines, exacerbating neuroinflammation, while M2 microglia play an anti-inflammatory role. In this context, Li et al., 2021b encapsulated interleukin-4 (IL-4) within the PPIX (sonosensitizer approved by FDA)-loaded liposomes to create an ultrasound-responsive nanomedicine. To enhance IS targeting ability, liposomes were coated with platelet membrane. Upon ultrasound irradiation, PPIX generates ROS, triggering peroxidation of the lipid bilayers, which facilitates the rapid release of IL-4. The localized accumulation of IL-4 in IS tissue induces the polarization of microglia towards the M2 phenotype, which ultimately serves a therapeutic role in neuroprotection. *In vivo* experiments performed on mouse with MCAO demonstrated that ultrasound-triggered liposomes showed significant protective effects on cerebral ischemic tissue after stroke.

## 4 Summary and prospect

Nanotechnology is currently utilized in various ways in the field of IS, including nanotechnology-assisted imaging diagnostics, targeted delivery of thrombolytic drugs to blood clots, and controlled drug release. Restoring blood flow and providing neuroprotection to ischemic brain tissue are crucial therapeutic strategies for IS. Administering effective neuroprotective treatment in a timely manner following a stroke or ischemic reperfusion injury can significantly enhance patients’ subsequent recovery and quality of life. However, the BBB poses a challenge to delivering neuroprotective agents to ischemic brain tissue at effective concentrations due to the short half-life and low bioavailability of drugs. Consequently, drug delivery methods for IS are gaining increasing attention in recent years.

Compared to traditional drug delivery methods, stimuli-responsive nanomedicines demonstrate higher efficiency in targeted delivery and better control over drug release, which enables more effective treatment while reducing the occurrence of adverse reactions such as cerebral hemorrhage. Stimuli-responsive nanomedicines generally involve endogenous stimuli response (ROS, enzymes, pH), exogenous stimuli response (light, magnetic field, ultrasound), and dual stimuli response. Many researchers have successfully designed and prepared nanocarriers with stimuli-responsive characteristics, including organic nanocarriers, inorganic nanocarriers, biomimetic nanocarriers, and composite nanocarriers. Organic nanomaterials are easily obtained, exhibit high biocompatibility, and are biodegradable *in vivo*. However, they have drawbacks such as poor stability and low encapsulation efficiency, which pose challenges for their practical clinical application in medicine. Biomimetic nanomaterials derived from red blood cells, immune cells, mesenchymal cells, and platelets demonstrate high biocompatibility, extended circulation time in the body, and enhanced efficiency in targeted delivery. Nevertheless, challenges in biomimetic nanocarrier research include low specificity in drug release and difficulty in large-scale production. Inorganic NPs possess photonic and magnetic responsiveness, making them more suitable for constructing exogenous stimuli-responsive nanomedicines. However, their potential toxicity and limited biocompatibility hinder the development prospects of inorganic nanomaterials. Studies have suggested that inorganic NPs can accumulate in neural tissue, but the potential long-term neurodegenerative effects remain unclear. In recent years, the combining of organic and inorganic NPs to form composite NPs has received significant attention from research teams due to their high biocompatibility, strong targeting ability, and low biological toxicity. This approach enables improved targeted drug delivery and specific drug release.

In spite of the great progress, there are still noticeable disparities between preclinical research and clinical applications in this field. The preparation of stimuli-responsive nanomedicines highlights their ability to target ischemic brain tissue, minimizing the impact on healthy tissue. However, the current assessment of safety and biocompatibility for stimuli-responsive nanomedicines is insufficient. Most studies provide data related to assessing drug toxicity to cells and tissues in animal models, but there is still a lack of research on stability, tissue compatibility, and immune response. In addition to short-term safety and biocompatibility assessments, more studies are necessary to evaluate the long-term safety of stimuli-responsive nanomedicines. These studies may include long-term animal experiments and clinical trials.

Furthermore, the complex pathophysiological processes involved in IS contribute to the apparent disparity between preclinical studies and clinical applications of stimuli-responsive nanomedicine delivery for IS treatment. This disparity arises from the necessity of conducting safety and biocompatibility assessments, optimizing the selection of drugs and carriers, and considering the intricacy and cost associated with large-scale clinical trials. In summary, long-term efforts are still needed to promote better development of stimuli-responsive nanomedicines in the field of anti-IS therapy.

## References

[B1] Albers G. W. Marks M. P. Kemp S. Christensen S. Tsai J. P. Ortega-Gutierrez S. (2018). Thrombectomy for stroke at 6 to 16 hours with selection by perfusion imaging. N. Engl. J. Med. 378 (8), 708–718. 10.1056/NEJMoa1713973 29364767 PMC6590673

[B2] Alkaff S. A. Radhakrishnan K. Nedumaran A. M. Liao P. Czarny B. (2020). Nanocarriers for stroke therapy: advances and obstacles in translating animal studies. Int. J. Nanomedicine 15, 445–464. 10.2147/ijn.S231853 32021190 PMC6982459

[B3] Arsava E. M. Helenius J. Avery R. Sorgun M. H. Kim G. M. Pontes-Neto O. M. (2017). Assessment of the predictive validity of etiologic stroke classification. JAMA Neurol. 74 (4), 419–426. 10.1001/jamaneurol.2016.5815 28241214 PMC5470360

[B4] Athanassiadis A. G. Ma Z. Moreno-Gomez N. Melde K. Choi E. Goyal R. (2022). Ultrasound-responsive systems as components for smart materials. Chem. Rev. 122 (5), 5165–5208. 10.1021/acs.chemrev.1c00622 34767350 PMC8915171

[B5] Berkhemer O. A. Fransen P. S. Beumer D. van den Berg L. A. Lingsma H. F. Yoo A. J. (2015). A randomized trial of intraarterial treatment for acute ischemic stroke. N. Engl. J. Med. 372 (1), 11–20. 10.1056/NEJMoa1411587 25517348

[B6] Cai X. Bandla A. Wang C. Liu Y.-H. Chuan C. K. Xu Y. (2022). Photothermal-activatable liposome carrying tissue plasminogen activator for photoacoustic image-guided ischemic stroke treatment. Small Struct. 3 (2), 2100118. 10.1002/sstr.202100118

[B7] Cao Z. Li S. Yang H. Xu C. Zhang Y. Yang X. (2021). Associations of behaviors, biological phenotypes and cardiovascular health with risks of stroke and stroke subtypes: a prospective cohort study. EClinicalMedicine 33, 100791. 10.1016/j.eclinm.2021.100791 33842871 PMC8020159

[B8] Chamorro Á. Dirnagl U. Urra X. Planas A. M. (2016). Neuroprotection in acute stroke: targeting excitotoxicity, oxidative and nitrosative stress, and inflammation. Lancet Neurol. 15 (8), 869–881. 10.1016/s1474-4422(16)00114-9 27180033

[B9] Chao B. H. Tu W. J. Wang L. D. (2021). Initial establishment of a stroke management model in China: 10 years (2011-2020) of stroke prevention project committee, national health commission. Chin. Med. J. Engl. 134 (20), 2418–2420. 10.1097/cm9.0000000000001856 34620751 PMC8654431

[B10] Cheng X. Xie Q. Sun Y. (2023). Advances in nanomaterial-based targeted drug delivery systems. Front. Bioeng. Biotechnol. 11, 1177151. 10.3389/fbioe.2023.1177151 37122851 PMC10133513

[B11] Cheng Y. Cheng A. Jia Y. Yang L. Ning Y. Xu L. (2021). pH-responsive multifunctional theranostic rapamycin-loaded nanoparticles for imaging and treatment of acute ischemic stroke. ACS Appl. Mater Interfaces 13 (48), 56909–56922. 10.1021/acsami.1c16530 34807583

[B12] Cheung J. Doerr M. Hu R. Sun P. Z. (2021). Refined ischemic penumbra imaging with tissue pH and diffusion kurtosis magnetic resonance imaging. Transl. Stroke Res. 12 (5), 742–753. 10.1007/s12975-020-00868-z 33159656 PMC8102648

[B13] Cook A. B. Decuzzi P. (2021). Harnessing endogenous stimuli for responsive materials in theranostics. ACS Nano 15 (2), 2068–2098. 10.1021/acsnano.0c09115 33555171 PMC7905878

[B14] Correa-Paz C. Navarro Poupard M. F. Polo E. Rodríguez-Pérez M. Taboada P. Iglesias-Rey R. (2019). *In vivo* ultrasound-activated delivery of recombinant tissue plasminogen activator from the cavity of sub-micrometric capsules. J. Control Release 308, 162–171. 10.1016/j.jconrel.2019.07.017 31310784

[B15] Cui W. Liu R. Jin H. Lv P. Sun Y. Men X. (2016). pH gradient difference around ischemic brain tissue can serve as a trigger for delivering polyethylene glycol-conjugated urokinase nanogels. J. Control Release 225, 53–63. 10.1016/j.jconrel.2016.01.028 26795685

[B16] Fan C. H. Ho Y. J. Lin C. W. Wu N. Chiang P. H. Yeh C. K. (2022). State-of-the-art of ultrasound-triggered drug delivery from ultrasound-responsive drug carriers. Expert Opin. Drug Deliv. 19 (8), 997–1009. 10.1080/17425247.2022.2110585 35930441

[B17] Fisher M. Savitz S. I. (2022). Pharmacological brain cytoprotection in acute ischaemic stroke - renewed hope in the reperfusion era. Nat. Rev. Neurol. 18 (4), 193–202. 10.1038/s41582-021-00605-6 35079135 PMC8788909

[B18] GBD 2019 Stroke Collaborators (2019). Global, regional, and national burden of stroke and its risk factors, 1990-2019: a systematic analysis for the Global Burden of Disease Study 2019. Lancet Neurol. 20 (10), 795–820. 10.1016/s1474-4422(21)00252-0 PMC8443449 34487721

[B19] George P. M. Steinberg G. K. (2015). Novel stroke therapeutics: unraveling stroke pathophysiology and its impact on clinical treatments. Neuron 87 (2), 297–309. 10.1016/j.neuron.2015.05.041 26182415 PMC4911814

[B20] Gerace E. Cialdai F. Sereni E. Lana D. Nosi D. Giovannini M. G. (2021). NIR laser photobiomodulation induces neuroprotection in an *in vitro* model of cerebral hypoxia/ischemia. Mol. Neurobiol. 58 (10), 5383–5395. 10.1007/s12035-021-02496-6 34319540 PMC8497317

[B21] Grayston A. Zhang Y. Garcia-Gabilondo M. Arrúe M. Martin A. Kopcansky P. (2022). Endovascular administration of magnetized nanocarriers targeting brain delivery after stroke. J. Cereb. Blood Flow. Metab. 42 (2), 237–252. 10.1177/0271678x211028816 34229512 PMC9122522

[B22] Gu X. Li Y. Chen S. Yang X. Liu F. Li Y. (2019). Association of lipids with ischemic and hemorrhagic stroke: a prospective cohort study among 267 500 Chinese. Stroke 50 (12), 3376–3384. 10.1161/strokeaha.119.026402 31658904

[B23] Guo X. Deng G. Liu J. Zou P. Du F. Liu F. (2018). Thrombin-responsive, brain-targeting nanoparticles for improved stroke therapy. ACS Nano 12 (8), 8723–8732. 10.1021/acsnano.8b04787 30107729

[B24] Harston G. W. Tee Y. K. Blockley N. Okell T. W. Thandeswaran S. Shaya G. (2015). Identifying the ischaemic penumbra using pH-weighted magnetic resonance imaging. Brain 138 (Pt 1), 36–42. 10.1093/brain/awu374 25564491 PMC4285197

[B25] Hassanpour S. Kim H. J. Saadati A. Tebon P. Xue C. van den Dolder F. W. (2020). Thrombolytic agents: nanocarriers in controlled release. Small 16 (40), e2001647. 10.1002/smll.202001647 32790000 PMC7702193

[B26] He W. Mei Q. Li J. Zhai Y. Chen Y. Wang R. (2021). Preferential targeting cerebral ischemic lesions with cancer cell-inspired nanovehicle for ischemic stroke treatment. Nano Lett. 21 (7), 3033–3043. 10.1021/acs.nanolett.1c00231 33755480

[B27] Herpich F. Rincon F. (2020). Management of acute ischemic stroke. Crit. Care Med. 48 (11), 1654–1663. 10.1097/ccm.0000000000004597 32947473 PMC7540624

[B28] Hill M. D. Goyal M. Menon B. K. Nogueira R. G. McTaggart R. A. Demchuk A. M. (2020). Efficacy and safety of nerinetide for the treatment of acute ischaemic stroke (ESCAPE-NA1): a multicentre, double-blind, randomised controlled trial. Lancet 395 (10227), 878–887. 10.1016/s0140-6736(20)30258-0 32087818

[B29] Hu J. Huang S. Zhu L. Huang W. Zhao Y. Jin K. (2018). Tissue plasminogen activator-porous magnetic microrods for targeted thrombolytic therapy after ischemic stroke. ACS Appl. Mater Interfaces 10 (39), 32988–32997. 10.1021/acsami.8b09423 30192506

[B30] Jian W. H. Wang H. C. Kuan C. H. Chen M. H. Wu H. C. Sun J. S. (2018). Glycosaminoglycan-based hybrid hydrogel encapsulated with polyelectrolyte complex nanoparticles for endogenous stem cell regulation in central nervous system regeneration. Biomaterials 174, 17–30. 10.1016/j.biomaterials.2018.05.009 29763775

[B31] Jin L. Zhu Z. Hong L. Qian Z. Wang F. Mao Z. (2023). ROS-responsive 18β-glycyrrhetic acid-conjugated polymeric nanoparticles mediate neuroprotection in ischemic stroke through HMGB1 inhibition and microglia polarization regulation. Bioact. Mater 19, 38–49. 10.1016/j.bioactmat.2022.03.040 35415314 PMC8980441

[B32] Jovin T. G. Li C. Wu L. Wu C. Chen J. Jiang C. (2022). Trial of thrombectomy 6 to 24 hours after stroke due to basilar-artery occlusion. N. Engl. J. Med. 387 (15), 1373–1384. 10.1056/NEJMoa2207576 36239645

[B33] Kelmanson I. V. Shokhina A. G. Kotova D. A. Pochechuev M. S. Ivanova A. D. Kostyuk A. I. (2021). *In vivo* dynamics of acidosis and oxidative stress in the acute phase of an ischemic stroke in a rodent model. Redox Biol. 48, 102178. 10.1016/j.redox.2021.102178 34773835 PMC8600061

[B34] Khizar S. Alrushaid N. Alam Khan F. Zine N. Jaffrezic-Renault N. Errachid A. (2023). Nanocarriers based novel and effective drug delivery system. Int. J. Pharm. 632, 122570. 10.1016/j.ijpharm.2022.122570 36587775

[B35] Kim H. Y. Kim T. J. Kang L. Kim Y. J. Kang M. K. Kim J. (2020). Mesenchymal stem cell-derived magnetic extracellular nanovesicles for targeting and treatment of ischemic stroke. Biomaterials 243, 119942. 10.1016/j.biomaterials.2020.119942 32179302

[B36] Leigh R. Knutsson L. Zhou J. van Zijl P. C. (2018). Imaging the physiological evolution of the ischemic penumbra in acute ischemic stroke. J. Cereb. Blood Flow. Metab. 38 (9), 1500–1516. 10.1177/0271678x17700913 28345479 PMC6125975

[B37] Li B. Chen R. Zhang Y. Zhao L. Liang H. Yan Y. (2019). RGD modified protein-polymer conjugates for pH-triggered targeted thrombolysis. ACS Appl. Bio Mater 2 (1), 437–446. 10.1021/acsabm.8b00644 35016307

[B38] Li C. Sun T. Jiang C. (2021a). Recent advances in nanomedicines for the treatment of ischemic stroke. Acta Pharm. Sin. B 11 (7), 1767–1788. 10.1016/j.apsb.2020.11.019 34386320 PMC8343119

[B39] Li M. Li J. Chen J. Liu Y. Cheng X. Yang F. (2020). Platelet membrane biomimetic magnetic nanocarriers for targeted delivery and *in situ* generation of nitric oxide in early ischemic stroke. ACS Nano 14 (2), 2024–2035. 10.1021/acsnano.9b08587 31927980

[B40] Li Y. Teng X. Yang C. Wang Y. Wang L. Dai Y. (2021b). Ultrasound controlled anti-inflammatory polarization of platelet decorated microglia for targeted ischemic stroke therapy. Angew. Chem. Int. Ed. Engl. 60 (10), 5083–5090. 10.1002/anie.202010391 33259112

[B41] Lin X. Li N. Tang H. (2022). Recent advances in nanomaterials for diagnosis, treatments, and neurorestoration in ischemic stroke. Front. Cell Neurosci. 16, 885190. 10.3389/fncel.2022.885190 35836741 PMC9274459

[B42] Liu B. Wu R. Gong S. Xiao H. Thayumanavan S. (2020). *In situ* formation of polymeric nanoassemblies using an efficient reversible click reaction. Angew. Chem. Int. Ed. Engl. 59 (35), 15135–15140. 10.1002/anie.202004017 32410309 PMC7666047

[B43] Liu H. Sun R. Wang L. Chen X. Li G. Cheng Y. (2022a). Biocompatible iron oxide nanoring-labeled mesenchymal stem cells: an innovative magnetothermal approach for cell tracking and targeted stroke therapy. ACS Nano 16 (11), 18806–18821. 10.1021/acsnano.2c07581 36278899

[B44] Liu M. Yang M. Wan X. Tang Z. Jiang L. Wang S. (2022b). From nanoscopic to macroscopic materials by stimuli-responsive nanoparticle aggregation. Adv. Mater 35, e2208995. 10.1002/adma.202208995 36409139

[B45] Lu Y. Li C. Chen Q. Liu P. Guo Q. Zhang Y. (2019). Microthrombus-targeting micelles for neurovascular remodeling and enhanced microcirculatory perfusion in acute ischemic stroke. Adv. Mater 31 (21), e1808361. 10.1002/adma.201808361 30957932

[B46] Luo L. Zang G. Liu B. Qin X. Zhang Y. Chen Y. (2021). Bioengineering CXCR4-overexpressing cell membrane functionalized ROS-responsive nanotherapeutics for targeting cerebral ischemia-reperfusion injury. Theranostics 11 (16), 8043–8056. 10.7150/thno.60785 34335979 PMC8315061

[B47] Lv W. Xu J. Wang X. Li X. Xu Q. Xin H. (2018). Bioengineered boronic ester modified dextran polymer nanoparticles as reactive oxygen species responsive nanocarrier for ischemic stroke treatment. ACS Nano 12 (6), 5417–5426. 10.1021/acsnano.8b00477 29869497

[B48] Ma H. Jiang Z. Xu J. Liu J. Guo Z. N. (2021). Targeted nano-delivery strategies for facilitating thrombolysis treatment in ischemic stroke. Drug Deliv. 28 (1), 357–371. 10.1080/10717544.2021.1879315 33517820 PMC8725844

[B49] Marko M. Miksova D. Ebner J. Lang M. Serles W. Sommer P. (2022). Temporal trends of functional outcome in patients with acute ischemic stroke treated with intravenous thrombolysis. Stroke 53 (11), 3329–3337. 10.1161/strokeaha.121.038400 36000395

[B50] Mei T. Kim A. Vong L. B. Marushima A. Puentes S. Matsumaru Y. (2019). Encapsulation of tissue plasminogen activator in pH-sensitive self-assembled antioxidant nanoparticles for ischemic stroke treatment - synergistic effect of thrombolysis and antioxidant. Biomaterials 215, 119209. 10.1016/j.biomaterials.2019.05.020 31181394

[B51] Parvez S. Kaushik M. Ali M. Alam M. M. Ali J. Tabassum H. (2022). Dodging blood brain barrier with "nano" warriors: novel strategy against ischemic stroke. Theranostics 12 (2), 689–719. 10.7150/thno.64806 34976208 PMC8692911

[B52] Pawlowski C. L. Li W. Sun M. Ravichandran K. Hickman D. Kos C. (2017). Platelet microparticle-inspired clot-responsive nanomedicine for targeted fibrinolysis. Biomaterials 128, 94–108. 10.1016/j.biomaterials.2017.03.012 28314136 PMC6526940

[B53] Qin C. Yang S. Chu Y. H. Zhang H. Pang X. W. Chen L. (2022). Signaling pathways involved in ischemic stroke: molecular mechanisms and therapeutic interventions. Signal Transduct. Target Ther. 7 (1), 215. 10.1038/s41392-022-01064-1 35794095 PMC9259607

[B54] Rahoui N. Jiang B. Taloub N. Huang Y. D. (2017). Spatio-temporal control strategy of drug delivery systems based nano structures. J. Control Release 255, 176–201. 10.1016/j.jconrel.2017.04.003 28408201

[B55] Refaat A. Del Rosal B. Palasubramaniam J. Pietersz G. Wang X. Moulton S. E. (2021). Near-infrared light-responsive liposomes for protein delivery: towards bleeding-free photothermally-assisted thrombolysis. J. Control Release 337, 212–223. 10.1016/j.jconrel.2021.07.024 34284049

[B56] Ruscu M. Cercel A. Kilic E. Catalin B. Gresita A. Hermann D. M. (2023). Nanodrugs for the treatment of ischemic stroke: a systematic review. Int. J. Mol. Sci. 24 (13), 10802. 10.3390/ijms241310802 37445979 PMC10341504

[B57] Saravanakumar G. Kim J. Kim W. J. (2017). Reactive-oxygen-species-responsive drug delivery systems: promises and challenges. Adv. Sci. (Weinh) 4 (1), 1600124. 10.1002/advs.201600124 28105390 PMC5238745

[B58] Shao J. Abdelghani M. Shen G. Cao S. Williams D. S. van Hest J. C. M. (2018). Erythrocyte membrane modified Janus polymeric motors for thrombus therapy. ACS Nano 12 (5), 4877–4885. 10.1021/acsnano.8b01772 29733578 PMC5968433

[B59] Spitzer D. Guérit S. Puetz T. Khel M. I. Armbrust M. Dunst M. (2022). Profiling the neurovascular unit unveils detrimental effects of osteopontin on the blood-brain barrier in acute ischemic stroke. Acta Neuropathol. 144 (2), 305–337. 10.1007/s00401-022-02452-1 35752654 PMC9288377

[B60] Su Y. Guo C. Chen Q. Guo H. Wang J. Kaihang M. (2022). Novel multifunctional bionanoparticles modified with sialic acid for stroke treatment. Int. J. Biol. Macromol. 214, 278–289. 10.1016/j.ijbiomac.2022.06.102 35716787

[B61] Teng Y. Jin H. Nan D. Li M. Fan C. Liu Y. (2018). *In vivo* evaluation of urokinase-loaded hollow nanogels for sonothrombolysis on suture embolization-induced acute ischemic stroke rat model. Bioact. Mater 3 (1), 102–109. 10.1016/j.bioactmat.2017.08.001 29744447 PMC5935765

[B62] Tiedt S. Buchan A. M. Dichgans M. Lizasoain I. Moro M. A. Lo E. H. (2022). The neurovascular unit and systemic biology in stroke - implications for translation and treatment. Nat. Rev. Neurol. 18 (10), 597–612. 10.1038/s41582-022-00703-z 36085420

[B63] Tsivgoulis G. Katsanos A. H. Sandset E. C. Turc G. Nguyen T. N. Bivard A. (2023). Thrombolysis for acute ischaemic stroke: current status and future perspectives. Lancet Neurol. 22, 418–429. 10.1016/s1474-4422(22)00519-1 36907201

[B64] Tu W. J. Zhao Z. Yin P. Cao L. Zeng J. Chen H. (2023). Estimated burden of stroke in China in 2020. JAMA Netw. Open 6 (3), e231455. 10.1001/jamanetworkopen.2023.1455 36862407 PMC9982699

[B65] Tuo Q. Z. Liu Y. Xiang Z. Yan H. F. Zou T. Shu Y. (2022a). Thrombin induces ACSL4-dependent ferroptosis during cerebral ischemia/reperfusion. Signal Transduct. Target Ther. 7 (1), 59. 10.1038/s41392-022-00917-z 35197442 PMC8866433

[B66] Tuo Q. Z. Zhang S. T. Lei P. (2022b). Mechanisms of neuronal cell death in ischemic stroke and their therapeutic implications. Med. Res. Rev. 42 (1), 259–305. 10.1002/med.21817 33957000

[B67] Walter K. (2022). What is acute ischemic stroke? Jama 327 (9), 885. 10.1001/jama.2022.1420 35230392

[B68] Wang P. Gong Q. Hu J. Li X. Zhang X. (2021). Reactive oxygen species (ROS)-Responsive prodrugs, probes, and theranostic prodrugs: applications in the ROS-related diseases. J. Med. Chem. 64 (1), 298–325. 10.1021/acs.jmedchem.0c01704 33356214

[B69] Wang Y. J. Li Z. X. Gu H. Q. Zhai Y. Zhou Q. Jiang Y. (2022). China stroke statistics: an update on the 2019 report from the national center for healthcare quality management in neurological diseases, China national clinical research center for neurological diseases, the Chinese stroke association, national center for chronic and non-communicable disease control and prevention, Chinese center for disease control and prevention and institute for global neuroscience and stroke collaborations. Stroke Vasc. Neurol. 7 (5), 415–450. 10.1136/svn-2021-001374 35443985 PMC9614174

[B70] Wang Z. Zhao Y. Hou Y. Tang G. Zhang R. Yang Y. (2023). A thrombin-activated peptide-templated nanozyme for remedying ischemic stroke via thrombolytic and neuroprotective actions. Adv. Mater, e2210144. 10.1002/adma.202210144 36730098

[B71] Wu D. Zhou J. Zheng Y. Zheng Y. Zhang Q. Zhou Z. (2023). Pathogenesis-adaptive polydopamine nanosystem for sequential therapy of ischemic stroke. Nat. Commun. 14 (1), 7147. 10.1038/s41467-023-43070-z 37932306 PMC10628287

[B72] Wu S. Wu B. Liu M. Chen Z. Wang W. Anderson C. S. (2019). Stroke in China: advances and challenges in epidemiology, prevention, and management. Lancet Neurol. 18 (4), 394–405. 10.1016/s1474-4422(18)30500-3 30878104

[B73] Xiong Y. Wakhloo A. K. Fisher M. (2022). Advances in acute ischemic stroke therapy. Circ. Res. 130 (8), 1230–1251. 10.1161/circresaha.121.319948 35420919

[B74] Xu J. Wang X. Yin H. Cao X. Hu Q. Lv W. (2019). Sequentially site-specific delivery of thrombolytics and neuroprotectant for enhanced treatment of ischemic stroke. ACS Nano 13 (8), 8577–8588. 10.1021/acsnano.9b01798 31339295

[B75] Yang H. Luo Y. Hu H. Yang S. Li Y. Jin H. (2021). pH-sensitive, cerebral vasculature-targeting hydroxyethyl starch functionalized nanoparticles for improved angiogenesis and neurological function recovery in ischemic stroke. Adv. Healthc. Mater 10 (12), e2100028. 10.1002/adhm.202100028 34028998

[B76] Yang P. Zhang Y. Zhang L. Zhang Y. Treurniet K. M. Chen W. (2020). Endovascular thrombectomy with or without intravenous Alteplase in acute stroke. N. Engl. J. Med. 382 (21), 1981–1993. 10.1056/NEJMoa2001123 32374959

[B77] Yang Q. Pu W. Hu K. Hu Y. Feng Z. Cai J. (2023). Reactive oxygen species-responsive transformable and triple-targeting butylphthalide nanotherapy for precision treatment of ischemic stroke by normalizing the pathological microenvironment. ACS Nano 17 (5), 4813–4833. 10.1021/acsnano.2c11363 36802489

[B78] Ye Q. Zhai F. Chao B. Cao L. Xu Y. Zhang P. (2022). Rates of intravenous thrombolysis and endovascular therapy for acute ischaemic stroke in China between 2019 and 2020. Lancet Reg. Health West Pac 21, 100406. 10.1016/j.lanwpc.2022.100406 35243459 PMC8873940

[B79] Yingze Y. Zhihong J. Tong J. Yina L. Zhi Z. Xu Z. (2022). NOX2-mediated reactive oxygen species are double-edged swords in focal cerebral ischemia in mice. J. Neuroinflammation 19 (1), 184. 10.1186/s12974-022-02551-6 35836200 PMC9281066

[B80] Yogendrakumar V. Beharry J. Churilov L. Alidin K. Ugalde M. Pesavento L. (2023). Tenecteplase improves reperfusion across time in large vessel stroke. Ann. Neurol. 93 (3), 489–499. 10.1002/ana.26547 36394101

[B81] Yoshimura S. Sakai N. Yamagami H. Uchida K. Beppu M. Toyoda K. (2022). Endovascular therapy for acute stroke with a large ischemic region. N. Engl. J. Med. 386 (14), 1303–1313. 10.1056/NEJMoa2118191 35138767

[B82] You Y. Liu Y. Ma C. Xu J. Xie L. Tong S. (2023). Surface-tethered ROS-responsive micelle backpacks for boosting mesenchymal stem cell vitality and modulating inflammation in ischemic stroke treatment. J. Control Release 362, 210–224. 10.1016/j.jconrel.2023.08.039 37619863

[B83] Yu H. Palazzolo J. S. Zhou J. Hu Y. Niego B. Pan S. (2022a). Bioresponsive polyphenol-based nanoparticles as thrombolytic drug carriers. ACS Appl. Mater Interfaces 14 (3), 3740–3751. 10.1021/acsami.1c19820 35019268

[B84] Yu Q. Jian Z. Yang D. Zhu T. (2022b). Perspective insights into hydrogels and nanomaterials for ischemic stroke. Front. Cell Neurosci. 16, 1058753. 10.3389/fncel.2022.1058753 36761147 PMC9902513

[B85] Yu W. Yin N. Yang Y. Xuan C. Liu X. Liu W. (2022c). Rescuing ischemic stroke by biomimetic nanovesicles through accelerated thrombolysis and sequential ischemia-reperfusion protection. Acta Biomater. 140, 625–640. 10.1016/j.actbio.2021.12.009 34902617

[B86] Yuan J. Li L. Yang Q. Ran H. Wang J. Hu K. (2021). Targeted treatment of ischemic stroke by bioactive nanoparticle-derived reactive oxygen species responsive and inflammation-resolving nanotherapies. ACS Nano 15 (10), 16076–16094. 10.1021/acsnano.1c04753 34606239

[B87] Zenych A. Fournier L. Chauvierre C. (2020). Nanomedicine progress in thrombolytic therapy. Biomaterials 258, 120297. 10.1016/j.biomaterials.2020.120297 32818824

[B88] Zhang H. Li Z. Dai Y. Guo E. Zhang C. Wang Y. (2019). Ischaemic stroke etiological classification system: the agreement analysis of CISS, SPARKLE and TOAST. Stroke Vasc. Neurol. 4 (3), 123–128. 10.1136/svn-2018-000226 31709117 PMC6812642

[B89] Zhang H. Qu H. He Q. Gao L. Zhang H. Wang Y. (2021). Thrombus-targeted nanoparticles for thrombin-triggered thrombolysis and local inflammatory microenvironment regulation. J. Control Release 339, 195–207. 10.1016/j.jconrel.2021.06.043 34214595

[B90] Zhang J. Li X. Kwansa H. Kim Y. T. Yi L. Hong G. (2017). Augmentation of poly(ADP-ribose) polymerase-dependent neuronal cell death by acidosis. J. Cereb. Blood Flow. Metab. 37 (6), 1982–1993. 10.1177/0271678x16658491 27381826 PMC5464694

[B91] Zhang L. Lin Z. Zeng L. Zhang F. Sun L. Sun S. (2022a). Ultrasound-induced biophysical effects in controlled drug delivery. Sci. China Life Sci. 65 (5), 896–908. 10.1007/s11427-021-1971-x 34453275

[B92] Zhang S. Peng B. Chen Z. Yu J. Deng G. Bao Y. (2022b). Brain-targeting, acid-responsive antioxidant nanoparticles for stroke treatment and drug delivery. Bioact. Mater 16, 57–65. 10.1016/j.bioactmat.2022.02.033 35386312 PMC8958421

[B93] Zhao W. Zhao Y. Wang Q. Liu T. Sun J. Zhang R. (2019). Remote light-responsive nanocarriers for controlled drug delivery: advances and perspectives. Small 15 (45), e1903060. 10.1002/smll.201903060 31599125

[B94] Zhao Y. Xie R. Yodsanit N. Ye M. Wang Y. Gong S. (2020). Biomimetic fibrin-targeted and H(2)O(2)-responsive nanocarriers for thrombus therapy. Nano Today 35, 100986. 10.1016/j.nantod.2020.100986 33072177 PMC7561002

[B95] Zhao Y. Z. Du L. N. Lu C. T. Jin Y. G. Ge S. P. (2013). Potential and problems in ultrasound-responsive drug delivery systems. Int. J. Nanomedicine 8, 1621–1633. 10.2147/ijn.S43589 23637531 PMC3635663

[B96] Zhuang J. Zhang X. Liu Q. Zhu M. Huang X. (2022). Targeted delivery of nanomedicines for promoting vascular regeneration in ischemic diseases. Theranostics 12 (14), 6223–6241. 10.7150/thno.73421 36168632 PMC9475455

